# Revisiting the nature of Cu sites in the activated Cu-SSZ-13 catalyst for SCR reaction†
†Electronic supplementary information (ESI) available: experimental section (sample description, *in situ* FTIR spectroscopy, synchrotron characterization, DFT-based analysis of XAS and XES data); XAS of hydrated Cu-SSZ-13; reversibility of the Cu(i) ↔ Cu(ii) redox chemistry and extra-ligand loss process upon high-temperature gas-flow switching; full report on DFT-optimized geometries; full report on EXAFS fitting results on O_2_-activated Cu-SSZ-13; EXAFS spectra for a low Cu-loading Cu-SSZ-13 sample after O_2_-activation at 400 °C. See DOI: 10.1039/c4sc02907k



**DOI:** 10.1039/c4sc02907k

**Published:** 2014-10-13

**Authors:** E. Borfecchia, K. A. Lomachenko, F. Giordanino, H. Falsig, P. Beato, A. V. Soldatov, S. Bordiga, C. Lamberti

**Affiliations:** a Department of Chemistry and INSTM Reference Center , University of Turin , via P. Giuria 7 , 10125 Turin , Italy . Email: carlo.lamberti@unito.it; b NIS Centre of Excellence , University of Turin , Italy; c Haldor Topsøe A/S , Nymøllevej 55, 2800 Kgs. , Lyngby , Denmark; d CrisDI Center of Crystallography , University of Turin , Italy; e Southern Federal University , Zorge street 5 , 344090 Rostov-on-Don , Russia

## Abstract

Cu-SSZ-13 is a highly active NH_3_-SCR catalyst for the abatement of harmful nitrogen oxides (NO_*x*_, *x* = 1, 2) from the exhausts of lean-burn engines. The study of Cu-speciation occurring upon thermal dehydration is a key step for the understanding of the enhanced catalytic properties of this material and for identifying the SCR active sites and their redox capability. Herein, we combined FTIR, X-ray absorption (XAS) and emission (XES) spectroscopies with DFT computational analysis to elucidate the nature and location of the most abundant Cu sites in the activated catalyst. Different Cu species have been found to be dominant as a function of the dehydration temperature and conditions. Data analysis revealed that the dehydration process of Cu cations is essentially completed at 250 °C, with the formation of dehydrated [CuOH]^+^ species hosted in close proximity to 1-Al sites in both d6r and 8r units of the SSZ-13 matrix. These species persist at higher temperatures only if a certain amount of O_2_ is present in the gas feed, while under inert conditions they undergo virtually total “self-reduction” as a consequence of an OH extra-ligand loss, resulting in bi-coordinated bare Cu^+^ cations. Synchrotron characterization supported by computational analysis allowed an unprecedented quantitative refinement of the local environment and structural parameters of these Cu(ii) and Cu(i) species.

## Abstract 2

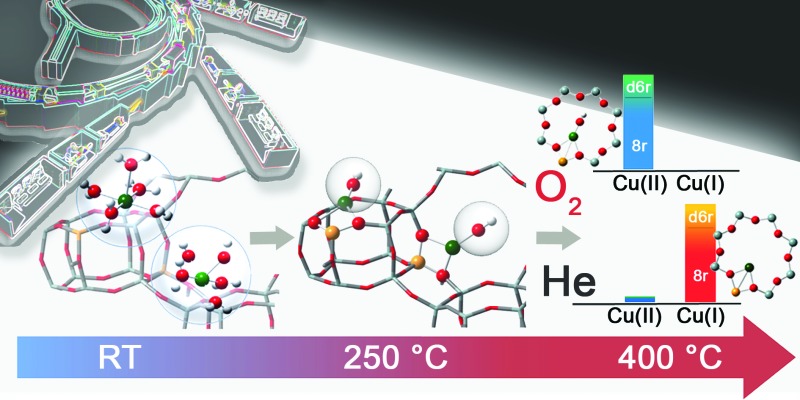
X-ray absorption and emission spectroscopy, FTIR and DFT unravel the major Cu species in the activated Cu-SSZ-13 catalyst for NH_3_-SCR.

## 1. Introduction

Selective catalytic reduction by NH_3_ (NH_3_-SCR) is considered to be one of the most efficient ways to remove environmentally harmful nitrogen oxides (NO_*x*_, *x* = 1, 2) from the exhausts of lean-burn engines.^1–4^ A variety of Cu-substituted zeolites have been investigated for NH_3_-SCR activity, and their stability in catalyst operating conditions has been examined. Early development efforts were focused on Cu-ZSM-5 and Cu-beta catalysts for their high activity over a wide range of conditions.^5,6^ Most recently, a small pore CHA-based material, *i.e.* Cu-exchanged SSZ-13, has become the subject of considerable study as it is now used commercially.^4,7–11^ Compared to Cu-ZSM-5 and Cu-beta, Cu-SSZ-13 has been found to be more active and selective, and less prone to deactivation by hydrocarbon inhibition or thermal degradation.^12^ Knowledge of the location and coordination of copper in the SSZ-13 zeolite is fundamental for fully understanding its superior SCR performances and for the clarification of the active species involved in the reaction mechanism.

SSZ-13 is a three-dimensional zeolite made up of double six rings (d6r), which are connected by four-membered ring units, and by 8-membered CHA composite units (8r). In the literature, most of the work agrees with the fact that Cu-SSZ-13 mainly contains monomeric Cu species which have been proposed as the active sites for NO_*x*_ reduction with NH_3_.^3^ Lobo and coworkers^13^ were the first to perform an XRD study on an O_2_-activated Cu-SSZ-13 catalyst (Si/Al = 6 and Cu/Al = 0.35), suggesting that Cu is only present as isolated Cu^2+^ ions, exclusively located in 6-membered ring windows, with a coordination number of 3 and average Cu–O distances of 2.2–2.3 Å. Korhonen *et al.* ^14^ employed UV-vis and EXAFS spectroscopy, supporting the location of Cu^2+^ ions on the plane of the d6r sub-units as the dominant species in an O_2_-activated Cu-SSZ-13 sample (Si/Al = 9 and Cu/Al = 0.18). They proposed that, after calcination, three framework oxygens coordinate to Cu^2+^ ions at an average distance of 1.93 Å.

Later, on the basis of TPR and FTIR results, Kwak *et al.* ^10^ observed another Cu ion location in SSZ-13 (Si/Al = 6) for Cu/Al > 0.2, which they assigned to isolated Cu^2+^ ions in the 8r. Subsequently, Gao *et al.* ^11^ concluded that for 0.2 ≤ Cu/Al ≤ 0.4 the active sites for ammonia oxidation were these isolated Cu ions in the 8r cage of SSZ-13. We recently found that the occurrence of isolated dehydrated [Cu^2+^(OH^–^)]^+^ species is also likely upon O_2_-activation of Cu-SSZ-13 (Si/Al = 13.1, Cu/Al = 0.44); from UV-vis analysis we also ruled out the presence of Cu–O–Cu dimeric species in SSZ-13. These species can instead readily form upon O_2_-activation in Cu-ZSM-5 and Cu-beta zeolites characterized by a similar Cu and Al content.^15^ Very recently, Andersen *et al.* ^16^ demonstrated the presence of significant amounts of Cu located at a specific crystallographic site in the 8r (∼80% of the Cu) by Rietveld/maximum entropy method (MEM) analysis applied to synchrotron powder X-ray diffraction data of dehydrated Cu-SSZ-13 (Si/Al = 15.5, Cu/Al = 0.45, similar to the sample investigated in our previous studies^15,17^ and in the present work). Based on DFT analysis, the authors assigned this site to [Cu^2+^(OH^–^)]^+^ complexes, preferentially stabilized in proximity of an isolated Al T-site in the 8r unit. The presence of a major fraction (∼50%) of Cu sites in activated Cu-SSZ-13, that exist as [Cu^2+^(OH^–^)]^+^ species which are EPR-silent due to a pseudo Jahn–Teller effect (PJTE), has been also recently proposed by Godiksen *et al.* ^18^ to explain the loss of EPR signal upon thermal dehydration at 250 °C.

In partial contrast with the findings reported above, Verma *et al.* ^19^ performed a combined experimental (UV-vis, XAS, catalytic measurements) and computational kinetic study of dry NO oxidation on Cu-SSZ-13 catalysts (Si/Al = 4.5), suggesting at least two Cu ion configurations within SSZ-13: for Cu/Al < 0.2, the dominant configuration should consist of dehydrated isolated Cu^2+^ ions, located in the vicinity of 2 framework Al sites, *i.e.* Al pairs, in the d6r units of SSZ-13. For Cu/Al = 0.2 all framework Al pairs in the d6r units are ion-exchanged by dehydrated isolated Cu^2+^ ions. For Cu/Al > 0.2, the authors suggested the occurrence of Cu_*x*_O_*y*_ species (per mole Cu, *x* ≥ 2, *y* ≥ 1), likely balanced by Al pairs in the 8-membered rings of SSZ-13.

From the scenario outlined above, it is clear that Cu speciation in the dehydrated SSZ-13 zeolite is greatly affected by the composition of the zeolite in terms of both Cu/Al and Si/Al atomic ratios. Unfortunately, Al distribution in zeolites is not uniform and, besides a few isolated exceptions,^20^ it has only been described from a statistical point of view. Even though DFT calculations have been successfully employed for a theoretical description of different Cu sites,^21,22^ a detailed experimental analysis on the nature, local environment and structural parameters of the dehydrated Cu species is still missing. Once the investigation of the NH_3_-SCR reaction mechanism is considered, the identification of the active sites of the catalyst is even more controversial. Indeed, several reaction cycles have been proposed so far,^4,7,23,24^ involving a variety of intermediate Cu species.

Herein we report a comprehensive FTIR, X-ray absorption (XAS)/emission (XES) and DFT study, with the aim of clarifying Cu speciation in a dehydrated Cu-SSZ-13 sample characterized by Si/Al = 12 and Cu/Al = 0.44, which is of foremost importance as a key substrate for reliably closing the SCR cycle and thus designing more efficient deNO_*x*_ catalysts. FTIR spectroscopy is particularly well suited for identifying the presence of specific species, and monitoring of the dehydration process *in situ*,^25,26^ being perfectly complemented by XAS spectroscopy. Indeed, XAS is an ideal tool for elucidating the average electronic properties and coordination geometry of the metal sites, which has been demonstrated to be greatly helpful in tackling the inherent complexity of the most efficient catalysts.^27–29^ When assisted by computational analysis, *in situ* XAS is indispensable for identifying the quantitatively-dominant Cu species formed in the catalyst after activation.^3,14,19,22,27,30–32^ XES has been proven to be an effective tool for local structure determination as well, since the probed high-lying valence orbitals are sensitive to chemical bonding.^28,29,33–35^ Being an independent element-selective method, it complements XAS data, making the combined analysis of XES, EXAFS and XANES more reliable. Notably, a combined *operando* XAS/XES approach supported by DFT analysis recently allowed Grunwaldt and co-workers to disclose the mechanism of the NH_3_-SCR over a Fe-ZSM-5 zeolite catalyst.^36^

Driven by these motivations, here we extend our recent combined *in situ* FTIR/XAS/XES and DFT study,^17^ which was primarily focused on the interaction of the catalyst with ammonia, by reporting a detailed investigation of activated Cu-SSZ-13, both in terms of final states and time/temperature-resolved evolution during the activation ramps. In addition, in this improved study we were able to conjugate XAS (in both the XANES and EXAFS regions) and XES analysis accessing combined information on oxidation state, density of occupied and unoccupied electronic states, local coordination geometry/symmetry and coordination number of Cu sites in the different conditions probed. Such a high level of information enabled a reliable DFT-assisted quantitative refinement of the Cu-environments most relevant in describing the average properties of activated Cu-SSZ-13.

Activation performed in an oxidant atmosphere (50% O_2_/He, herein referred to as O_2_-activation) is the most relevant to the real lean SCR conditions of the catalyst. However, by comparing the response of the catalyst to different activation atmospheres, such as pure He flow (hereinafter, He-activation), key information on the nature of the Cu sites can be obtained. In this respect, our investigation also provided novel insights into the mechanism of how Cu ions undergo the so called “self-reduction” upon He-activation, a key step which is crucial for the understanding of the Cu^2+^/Cu^+^ redox chemistry occurring under SCR conditions.

## 2. Results and discussion

### 2.1 Infrared characterization of activated Cu-SSZ-13: evidence for an OH “extra-ligand”

**Fig. 1:**
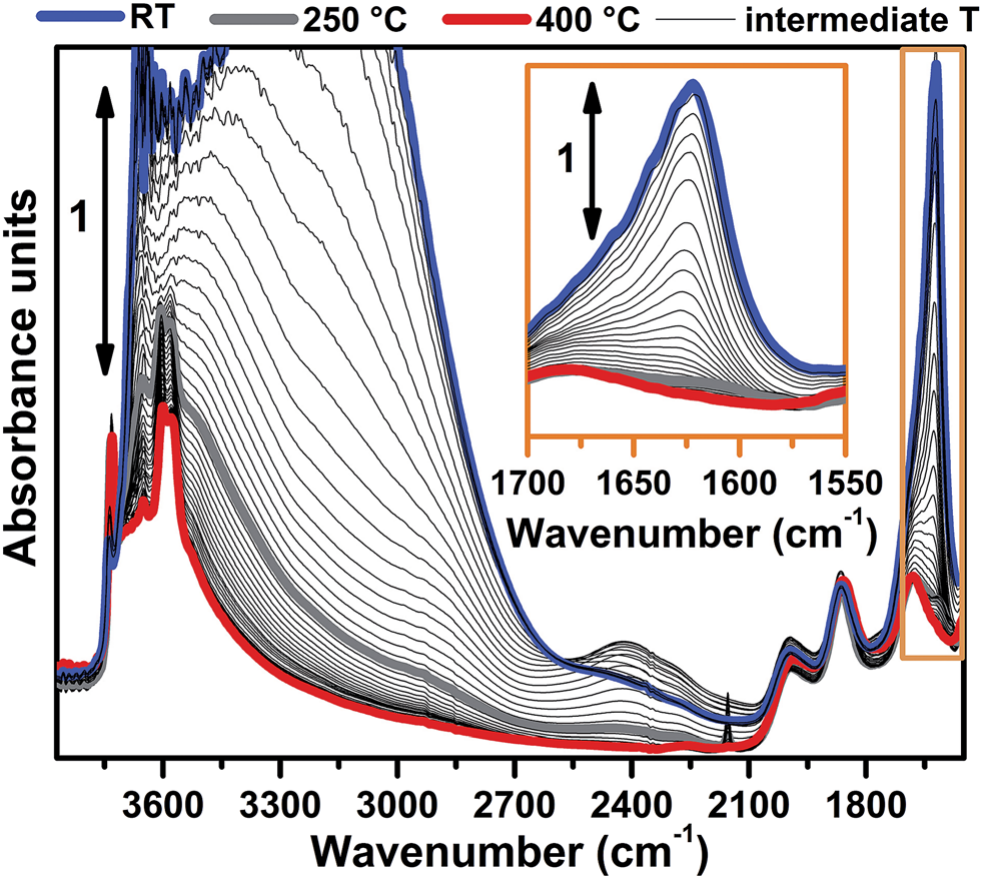
Dehydration of the Cu-SSZ-13 zeolite monitored by *in situ* FTIR spectroscopy. The sample was thermally treated by flowing 30 ml min^–1^ of 50% O_2_/He. Spectra colours refer to different temperatures: the bold blue curve to 30 °C, bold grey curve to 250 °C, bold red curve to 400 °C, thin black curves to intermediate temperatures. The inset shows a magnification of the signal related to the *δ*(OH) mode of molecularly adsorbed water (spectral region highlighted by the orange box in the main panel).

FTIR spectra collected at different temperatures upon Cu-SSZ-13 dehydration in 50% O_2_/He flow are reported in Fig. 1 (see ESI Sec. 1.2† for experimental details). Referring to the spectrum collected at 30 °C (blue curve), bands due to molecularly adsorbed water are observed at 1623 (*δ*), 3200 (*ν*_s_) and 3670 cm^–1^ (*ν*_as_).^37,38^ These bands can be associated to both water molecules physisorbed on zeolite channels and water molecules coordinated to copper sites as aquo-complexes. Broad features relating to H-bonded hydroxyl groups appear in the 3200–2100 cm^–1^ range, while the contribution of framework modes, *i.e.* overtones *ν*(T–O–T), gives rise to four low intensity bands at 1998, 1858, 1680 and 1535 cm^–1^. Notably, a *ν*(OH) contribution of silanols prevalently located on external surfaces of the zeolite is observed at 3737 cm^–1^, indicating that only a fraction of these species are perturbed by water molecules *via* H-bonds at 30 °C.

Spectral changes upon zeolite dehydration could be described as follows: (i) the gradual intensity decrease of the 3700, 3200, 3200–2100 cm^–1^ and 1623 cm^–1^ bands. The total disappearance of the band at 1623 cm^–1^ at 250 °C (grey curve) is a strong indication that molecular water is almost completely desorbed at this temperature (see also the magnification in the inset of Fig. 1). (ii) The gradual appearance of bands at 3611 and 3584 cm^–1^ is related to the *ν*(OH) of non H-bonded bridged hydroxyls groups with a strong Brønsted acidity.^15,39^

**Fig. 2:**
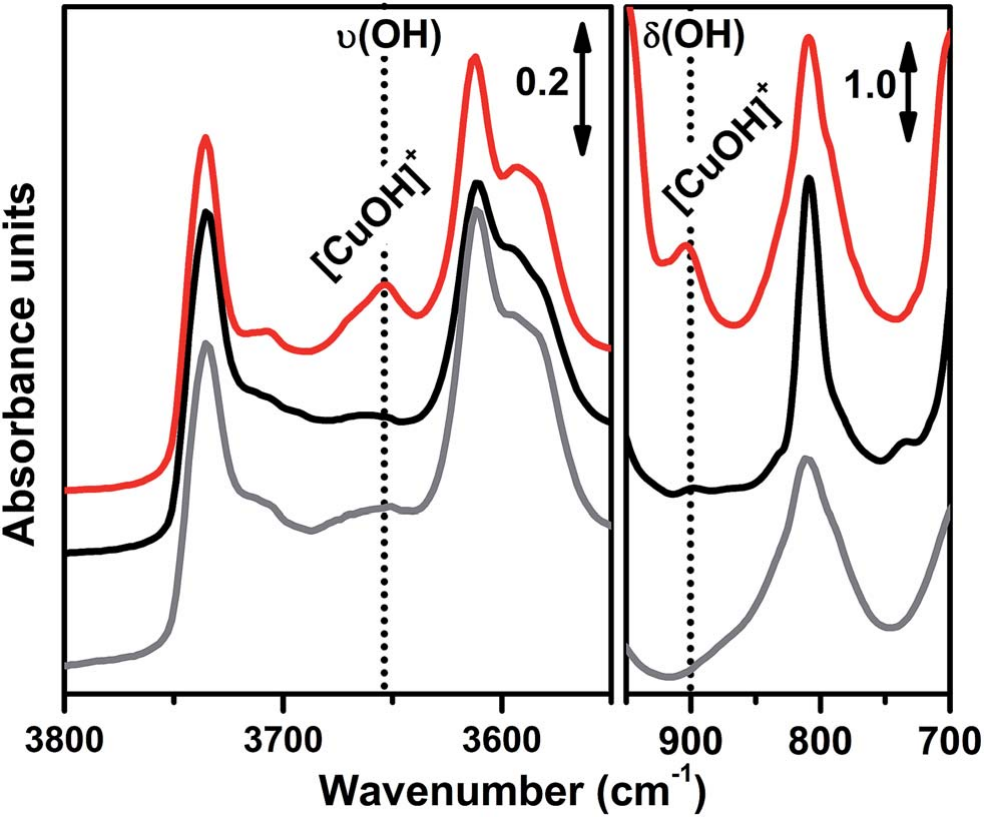
FTIR spectral evidence of the [CuOH]^+^ species in the O_2_-activated Cu-SSZ-13 zeolite (red curve). Spectra are reported in both the *ν*(OH) (left part) and *δ*(OH) (right part) regions. For the sake of comparison the spectra of He-activated Cu-SSZ-13 (black curve) and H-SSZ-13 (grey curve) zeolites are also reported; the spectra have been vertically shifted for clarity. In the Cu-SSZ-13 zeolite the bands relating to the [CuOH]^+^ species only appear upon O_2_-activation at 3656 and 905 cm^–1^.

In order to investigate the effects of different pre-treatments, Fig. 2 reports the FTIR spectra of the dehydrated Cu-SSZ-13 zeolite collected upon O_2_-activation and He-activation at 400 °C. For the sake of comparison the spectrum of the O_2_-activated H-SSZ-13 zeolite is also reported. All of the spectra show common bands related to the *ν*(O–H) modes of silanols (3737 cm^–1^) and Brønsted sites (3611 cm^–1^, 3584 cm^–1^).^15,39^ In addition, the spectrum of O_2_-activated Cu-SSZ-13 shows two distinct features at 3656 and 905 cm^–1^ which are not observed in the other cases. As we recently pointed out, the 3656 cm^–1^ feature only appears upon oxidative thermal treatment of the zeolite and it can be considered as a fingerprint of the [CuOH]^+^ species stabilized in the SSZ-13 matrix.^15^ On the basis of previous studies,^40^ the band at 905 cm^–1^ can be tentatively assigned to the *δ*(O–H) mode of the same species. It is important to note that a similar set of bands have already been observed for other metal containing zeolites, and they have been assigned to the corresponding M–OH species.^40–42^ The fact that these bands do not appear when the sample undergoes “self-reduction” upon He-activation (see black curve in Fig. 2) suggests that the stabilization of the OH extra-ligand on Cu(i) is unlikely.

Looking at the 3611 and 3584 cm^–1^ bands related to Brønsted sites, it is important to note that the intensity of these bands in dehydrated Cu-SSZ-13 is surprisingly high and only slightly lower compared to the parent material H-SSZ-13 (Fig. 2, grey curve). This is interesting since the intensity of these bands is expected to decrease consistently in a sample characterized by a Cu/Al ratio of 0.444. Indeed, it is generally assumed that when copper is introduced into the zeolite framework upon aqueous ion exchange, the positive charge (+2) of hydrated Cu^2+^ ions must be balanced by two negative charges, likely represented by two Al atoms in close proximity. Therefore, a ratio of Cu^2+^/Al^3+^ = 0.5 should represent the total ion exchange level. Conversely, our results clearly show that a considerable amount of non-exchanged sites, *i.e.* H^+^ of Brønsted sites, is still present even if the Cu^2+^/Al^3+^ ratio is not far from the stoichiometric exchange level. This evidence, together with the strong insights supporting the formation of [CuOH]^+^ as the dominant species upon O_2_-activation, can be explained according to two possible mechanisms. In those sites characterized by 2 Al atoms in close proximity (2Z^–^), the stabilization of divalent Cu^2+^(H_2_O)_*n*_ complexes upon the ion exchange procedure is favored; the gradual dehydration of these complexes leads to water dissociation to give [CuOH]^+^ and H^+^ species, eqn (1), where the latter is assumed to balance the charge of one of the two framework Al atoms. According to FTIR analysis (see Fig. 2), at 250 °C copper sites can be considered as fully dehydrated; for *T* > 250 °C the [CuOH]^+^ species can only be stabilized in an oxidative atmosphere, otherwise they undergo “self-reduction” as a consequence of OH extra-ligand loss. Alternatively, dehydration of Cu^2+^(H_2_O)_*n*_ complexes could lead to bare Cu^2+^ cations, eqn (1a). Conversely, in those sites characterized by only 1 Al (1Z^–^), the hydrated state upon aqueous ion exchange is likely represented by monovalent [Cu^2+^(H_2_O)_*n*_(OH)]^+^ complexes. In this case, the formation of [CuOH]^+^ upon dehydration does not require any water dissociation, eqn (2), and the concentration of Brønsted sites in the dehydrated material should be explained assuming that the total exchange level corresponds to [CuOH]^+^/Al^3+^ = 1. In all of these cases, the loss of the OH extra-ligand results in the reduction of the Cu^2+^ centre to Cu^+^.

1 2Z^–^: Cu^2+^(H_2_O)_*n*_ → Cu^2+^(H_2_O) + (*n* – 1)(H_2_O)^↑^ → [CuOH]^+^ + H^+^ → Cu^+^ + (OH)^↑^ + H^+^

1a 2Z^–^: Cu^2+^(H_2_O)_*n*_ → Cu^2+^ + *n*(H_2_O)^↑^

2 1Z^–^: [Cu^2+^(H_2_O)_*n*_(OH)]^+^ → [CuOH]^+^ + *n*(H_2_O)^↑^ → Cu^+^ + (OH)^↑^

The reversibility of the OH extra-ligand loss has been confirmed by XAS and FTIR spectroscopy, demonstrating that Cu^+^ sites rapidly undergo re-oxidation with consequent restoring of the [CuOH]^+^ species if they are exposed to a gas mixture of O_2_/H_2_O (see ESI Fig. S5a and b†).

### 2.2 Comparison between O_2_-activation and He-activation as monitored by XAS: final states and evolution

**Fig. 3:**
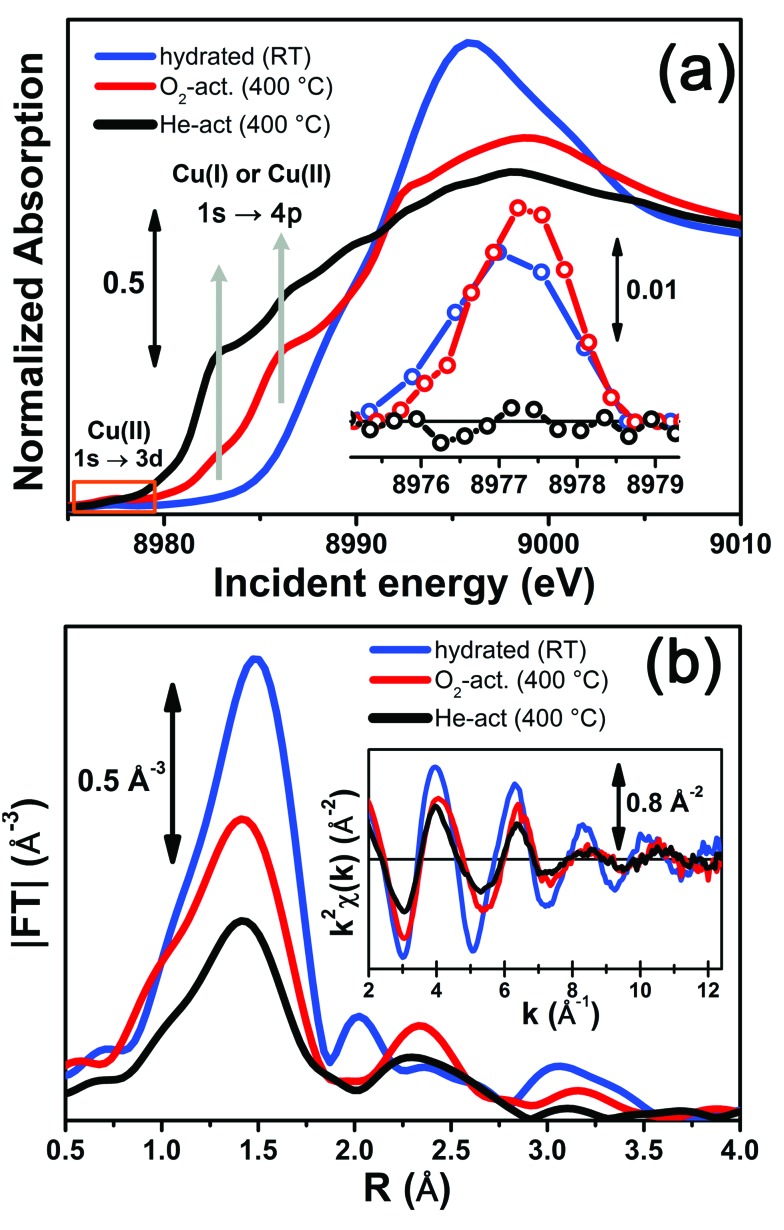
*In situ* static XAS data collected on the hydrated (RT), O_2_-activated and He-activated (400 °C) Cu-SSZ-13 catalyst. (a) Cu K-edge XANES spectra; the inset shows a magnification of the background-subtracted pre-edge peak highlighted by the orange box in the main panel. (b) Magnitude of the phase-uncorrected FT EXAFS spectra, obtained by transforming the *k* ^2^-weighted *χ*(*k*) curves reported in the inset in the (2.4–12.4) Å^–1^ range.

Fig. 3 reports a comparison between the XANES and EXAFS spectra collected for the initial (hydrated) and final states of the two activation processes, namely under O_2_-activation and He-activation conditions (see ESI Sec. 1.3† for experimental details). The characteristic XANES features occurring in the three probed states of the catalyst are the same as those observed in our previous study,^17^ where they have been assigned on the basis of the broad literature on Cu K-edge XANES in metal-exchanged zeolites^22,43–49^ and other systems.^50,51^

As expected from previous investigations by us^17^ and others,^22,30,32^ both the XANES and EXAFS spectra of the hydrated material (Fig. 3a and b, respectively, blue lines) closely resemble the spectra collected on a Cu(ii)-acetate aqueous solution (see ESI Fig. S4†), where no significant differences can be identified within the available data quality. The structure of hydrated Cu(ii) ions has been thoroughly analyzed in previous XAS studies^52–55^ and, despite being an elementary case in chemistry, it represents an ongoing challenge for EXAFS and XANES analysis. In particular, the most recent reports suggest a dynamic equilibrium between Cu(ii) sites 6-, 5- and even 4-coordinated to O atoms from H_2_O molecules. A preferred configuration was hardly distinguished even with the most sophisticate XANES analysis approaches.^54,55^ The similarity in the XAS spectra demonstrates that equivalent conditions are also locally verified in hydrated Cu-SSZ-13, crowded with highly mobile Cu(ii) aquo-complexes, resulting in a first-shell magnitude of the |FT[*k* ^2^ *χ*(*k*)]| spectrum compatible with time-averaged coordination to 5 O ligands. Here, no evidence of interaction with the zeolite framework is observed, due to the absence of well-defined coordination shells at higher *R*-distances.^17,22,30,32^ Unfortunately, it is impossible to distinguish by XAS the [Cu(H_2_O)_*n*–1_(OH)]^+^ and [Cu(H_2_O)_*n*_]^2+^ complexes (with *n* ranging from 4 to 6), due to the weak scattering amplitude of H.

O_2_-activation resulted in a XANES spectrum fully in line with that previously reported by us^17^ and others,^14,22^ (Fig. 3a, red line) pointing out the effectiveness of an oxidant atmosphere in the inhibition of the “self reduction” effect^48,56^ during Cu-SSZ-13 activation. The XANES signature of the O_2_-activated catalyst is typical of Cu(ii) sites in a less coordinated environment, characterized by a lower symmetry^48,51,56,57^ with respect to that associated to the hydrated material.

Conversely, He-activation resulted in the typical XANES spectrum of Cu(i) sites in non-linear, low-coordination number configurations,^50,58,59^ with a prominent and highly-structured pre-edge region developing from ∼8982 eV, consistent with that observed upon *in vacuo* activation in our previous study.^17^ Notably, in the present experiment we achieved a more complete reduction of Cu centres with respect to the *in vacuo* activation, by prolonged waiting in the He flow at 400 °C. Indeed, no detectable peak corresponding to the 1s → 3d transition, which fingerprints the presence of Cu(ii),^43,44,48,50,51,56,59^ was observed in the background-subtracted XANES spectrum of He-activated Cu-SSZ-13 (see Fig. 3a, inset). A residual signal from this peak after activation in He was visible only in high energy resolution fluorescence detected (HERFD) XANES spectra, due to the significantly decreased background in the pre-edge region (see experimental spectrum in Fig. 10b). Presumably, it originates from small fractions (indicatively ≤10% at. Cu for each species) of still-oxidized Cu species, such as isolated Cu(ii) sites (most likely in the d6r, charge-balanced by 2 Al atoms), CuO nano-clusters or Cu–O–Cu dimers, as also recently evidenced by EPR^18^ and XRD^16^ studies on Cu-SSZ-13 samples with equivalent Si/Al and Cu/Al ratios. Being structurally flexible, inhomogeneous and minor, the latter multi-nuclear Cu species may escape detection by EXAFS, despite the presence of high-amplitude metal–metal scattering paths.

Very interestingly, EXAFS shows a substantial lowering of the first shell magnitude after both activations with respect to the hydrated state (see Fig. 3b), which is clearly more pronounced for the He-activated sample. This novel evidence is crucial for correlating the Cu(ii) → Cu(i) reduction to the “extra-ligand” loss mechanism suggested by FTIR results, which is promoted or inhibited depending on the atmosphere, either oxidant or inert, in which the thermal treatment is conducted. Moreover, EXAFS evidences the interaction of Cu centres with the framework after both activations, with the appearance of a well-defined second coordination shell in the 2–3 Å region of the phase uncorrected |FT[*k* ^2^ *χ*(*k*)]| spectra. EXAFS fits reported in the following confirmed this assignment, indicating a major contribution from the single scattering (SS) paths involving the nearest neighbour T (T = Al, Si) atom(s) around the Cu sites in the d6r and 8r units of the SSZ-13 zeolite.

**Fig. 4:**
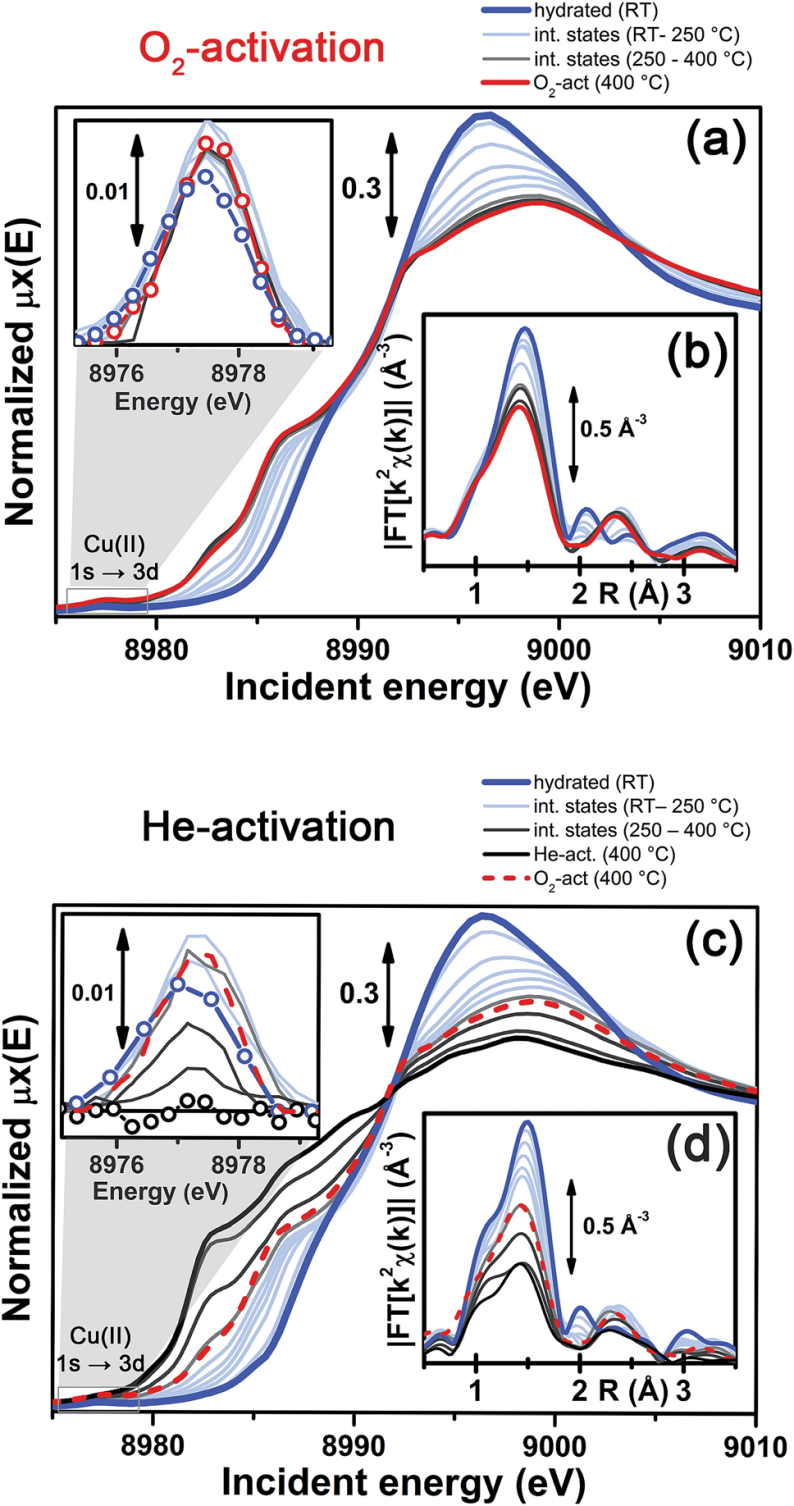
*In situ* temperature-dependent XAS data collected during O_2_-activation and He-activation processes. (a and b) XANES spectra, part (a), and magnitude of the FT of the *k* ^2^-weighted *χ*(*k*) EXAFS curves, performed in the (2.4–12.4) Å^–1^ range, part (b), measured during O_2_-activation of the Cu-SSZ-13 catalyst. The inset of part (a) shows the magnified background-subtracted Cu(ii) 1s → 3d pre-edge peak highlighted by the grey box in the main panel, for selected intermediate temperature steps. (c and d) As for parts (a and b) but for He-activation of the Cu-SSZ-13 catalyst. The final spectrum collected on the O_2_-activated Cu-SSZ-13 sample at 400 °C is also reported for comparison in panels (c and d).

More detailed insights can be obtained by monitoring *in situ* the evolution of the XANES and EXAFS spectra collected during the O_2_-activation and He-activation ramps, summarized in Fig. 4. We will first focus on the O_2_-activation process (Fig. 4a and b), and then discuss the additional modifications observed during activation in the He flow (Fig. 4c and d).

The evolution of the XANES features (Fig. 4a) is consistent with a progressive dehydration process, where the water molecules in the Cu(ii) hydration sphere are progressively removed, as evidenced by the decrease of the intense white line characteristic of the hydrated material. Simultaneously, the partially dehydrated Cu(ii) ions adopt specific positions in the framework, in a less symmetric coordination environment with respect to the hydrated conditions. The lowering in coordination symmetry upon dehydration is reflected by the development of a pre-edge shoulder assigned to 1s → 4p transitions in the 8985–8990 eV range, typical of Cu(ii) sites.^48,56,60^ In addition, the Cu(ii) fingerprint peak at *ca.* 8977.5 eV slightly grows in intensity with increasing temperature, which is also consistent with a less symmetric Cu coordination geometry. The evolution of the XANES features during O_2_-activation is qualitatively in agreement with what has been recently reported by Kwak *et al.* ^32^ for Cu-SSZ-13 (Si/Al = 6, Cu/Al = 0.4). Combining XANES, TPD-XRD and *in situ* DRIFT measurements, the authors confirmed that upon dehydration Cu^2+^ ions migrate into cationic positions where they strongly interact with the zeolite framework, although they did not quantitatively test specific structural environments for the activated Cu centres. Aiming for more detailed structural insights, we will use EXAFS to determine the dominant coordination environment of the metal, and how it evolves during activation, as a practical way for structural refinement.

In particular, the FT EXAFS spectra collected on our Cu-SSZ-13 sample during O_2_-activation (Fig. 4b) shows a progressive decrease of the first coordination shell, resulting from Cu–O single scattering (SS) paths (qualitatively from an oxygen coordination number *N*_O_ ∼5 to *N*_O_ ∼3, see below for quantitative fitting). The reduction in the first shell amplitude is accompanied by the modifications of the signal in the 2–3 Å region, where the unstructured signal characteristic of the hydrated material (mainly resulting from multiple scattering (MS) paths associated with linear O–Cu–O configurations involving the O(H_2_O) ligands in the equatorial plane of the aquo-complex^53^) is progressively replaced by the well-defined coordination shell associated with increased interaction with the framework.

It is important to note that, for the O_2_-activated material (Fig. 4a and b), for both the XANES and EXAFS spectra, the major evolution is observed in the RT–250 °C temperature range, whereas the spectral features are only slightly modified by increasing the temperature up to 400 °C. Here, the small intensity loss of the EXAFS FT signal is due only to the increase of the Debye–Waller (DW) factors with temperature. Interestingly, the FTIR data reported in Fig. 1 clearly show that at 250 °C no more adsorbed molecular water is present in the system: the dehydration of the Cu(ii) centres is thus completed, and the metal ions are likely already set in well-defined positions in the zeolite units.

In contrast to what was observed in an oxidant atmosphere, the evolution of the XANES and EXAFS features during He-activation can be rationalized only by considering two distinct steps. The first step, which we will refer to as dehydration, occurs from RT to ∼250 °C, while from 250 °C up to 400 °C we identified a reduction step.

The dehydration step is fully equivalent to what was observed during O_2_-activation. The XANES and EXAFS spectra collected at the final state of the O_2_-activation are reported for comparison as red dashed lines in Fig. 4c and d, respectively: it can be clearly observed that the final state obtained upon stabilization in O_2_/He flow at 400 °C is equivalent to the intermediate state reached at ∼270 °C in He flow.

The evolution of the system during activation in the 250–400 °C range is then dramatically different depending on the atmosphere in which the process is conducted. In particular, in the case of O_2_-activation we observed a stabilization of the spectral features, while for activation in a He flow, the XANES features showed pronounced modifications as a function of the temperature (Fig. 4c), including the development of pre-edge peaks in the 8982–8990 eV range, with intensities and energy positions associated with Cu(i) species. Simultaneously, a steep decrease in the intensity of the Cu(ii) fingerprint peak at ∼8977.5 eV is observed, which unambiguously demonstrates the Cu(ii) → Cu(i) reduction, involving progressively more and more Cu sites as the temperature increases, resulting in virtually complete erosion of the peak.

Importantly, during the reduction step EXAFS spectra showed a further decrease in the first-shell magnitude with respect to the final state reached after O_2_-activation (Fig. 4d), consistent with the loss of one of about three oxygen ligands in the first coordination shell of the Cu sites being reduced once completely dehydrated.

In summary, the above presented combined XANES and EXAFS evidence is in full agreement with the FTIR results, and strongly supports the formation of Z^–^[CuOH]^+^ complexes at the end of the dehydration step up to *ca.* 250 °C, according to the routes proposed in eqn (1) and (2). The stabilization of these species in the proximity of 1Z^–^ sites, balancing the charge of the [CuOH]^+^ complex, is highly favored during oxidative thermal treatment. Conversely, when the dehydrated catalyst is further heated in an inert atmosphere, Cu(ii) centres gradually undergo reduction, which likely occurs *via* homolytic cleavage of the Cu–OH bond^18,46^ and loss of the OH “extra-ligand”, leaving “bare” Cu(i) sites in their pristine framework location. Notably, these sites showed a strong propensity toward re-oxidation once the gas flow is switched from pure He to an O_2_/He + 5% H_2_O mixture. As shown in ESI Fig. S5a,† under these conditions at 400 °C the XAS signature of the final Cu(ii) state observed at the end of the O_2_-activation was quickly restored. Following the same experiment by FTIR spectroscopy, the reappearance of the [CuOH]^+^ fingerprint band at 3656 cm^–1^ is readily observed (see ESI Fig. S5b†).^15^ These results reinforce the evidence for a direct connection between the oxidation state of the Cu centres and the coordination of an extra-ligand group. Contextually, it is important to emphasize that the extra-ligand loss with the consequent reduction of the [CuOH]^+^ species can also occur in the dehydrated material: Fig. S5c in the ESI† clearly shows that Cu(i) XAS features were gradually restored if the O_2_-activated material, *i.e.* one containing a high fraction of the [CuOH]^+^ species, is contacted with a He flow at 400 °C. This result is in contrast with the belief that Cu sites could be self-reduced by carbonaceous deposits left in the zeolite during the preparation and/or by hydrocarbon impurities adsorbed from the atmosphere.^61^ Indeed, this kind of species is not expected to persist upon an O_2_-activation procedure at 400 °C.

Having these considerations in mind, in the following sections we will investigate in more detail the coordination environments of Cu(ii) and Cu(i) ions in Cu-SSZ-13 by DFT, on the basis of the principal configurations proposed in the literature. Hence, the resulting candidate structures will be systematically tested by EXAFS fits and XANES/XES simulations, aiming to achieve the best reproduction of the X-ray absorption and emission spectra collected on the catalyst after O_2_-activation and He-activation.

### 2.3 DFT-based modeling of the possible Cu(ii) and Cu(i) sites in activated Cu-SSZ-13

**Fig. 5:**
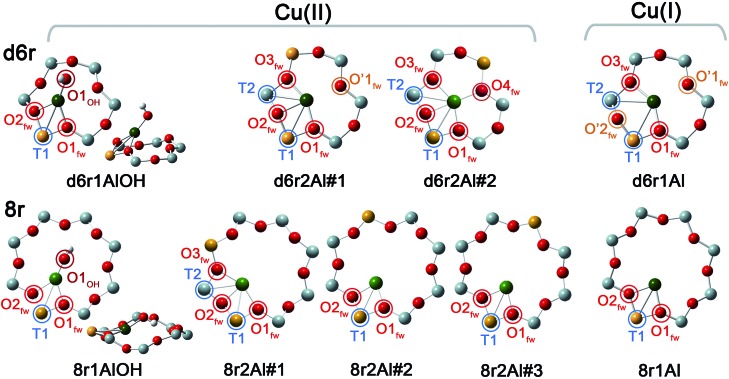
DFT-optimized structures highlighting the local coordination environment of Cu(ii) and Cu(i) sites in the d6r and 8r units of the CHA framework. Atom colour code: Cu, green; H, white; O, red; Al, gold; Si, gray. Coloured circles highlight the principal shells of atomic neighbours surrounding the Cu centres, for which average bond distances are reported in Table 1.

In our computational screening (see ESI Sec. 1.4.1† for details), we considered the two principal locations for the Cu ions in the CHA framework which were proposed in previous theoretical^21,22^ and experimental studies,^3,4,13,31^ *i.e.* the planes of the d6r and 8r. Hence, we envisaged two possible configurations for balancing the charge of Cu^2+^ and Cu^+^ centres, resulting from O_2_-activation and He-activation as clearly shown by XANES. In particular, we considered: (i) only the charge-compensating effect of Al atoms located in the same unit in which Cu is hosted or (ii) the combination of the effect of the Al atoms in the proximity of Cu sites and the presence of an (OH)^–^ extra-ligand coordinated to the Cu centre. The resulting DFT-optimized geometries, accounting for all of the possible configurations according to Löwenstein's rule,^62^ are reported in Fig. 5.

Under the previous assumptions, Cu(ii) sites could be obtained by stabilizing a [CuOH]^+^ complex in the proximity of a 1Z^–^ site (d6r1AlOH and 8r1AlOH models in Fig. 5) or hosting a “bare” Cu^2+^ cation next to a 2Z^–^ site (d6r2Al#1, 2 and 8r2Al#1–3 models in Fig. 5). Conversely, for Cu(i) sites only the configuration with a “bare” Cu^+^ cation next to a 1Z^–^ site is available, both in the d6r and 8r units (d6r1Al and 8r1Al models in Fig. 5). For additional explanation of the nomenclature adopted for the different DFT models see ESI Sec. 4, Scheme S1.† The average bond distances for the principal shells of atomic neighbours surrounding the Cu centres observed for all of the DFT-optimized geometries are reported in Table 1, whereas a complete report on the individual bond lengths and a detailed description of the different configurations can be found in ESI Sec. 4.†

**Table 1:** DFT-optimized average bond distances from Cu and coordination numbers for the principal shells of atomic neighbours surrounding Cu(ii) and Cu(i) centres in the d6r and 8r units of the CHA framework. See Fig. 5 for the atoms labeling code

Model parameters	Cu(ii) sites models							Cu(i) sites models	
	d6r1AlOH	d6r2Al#1	d6r2Al#2	8r1AlOH	8r2Al#1	8r2Al#2	8r2Al#3	d6r1Al	8r1Al
*N*_OH_	1			1					
Cu–O_OH_ (Å)	1.774	—	—	1.757	—	—	—	—	—
*N*_O(fw)_	2	3	4	2	3	2	2	2	2
Cu–O_fw_ (Å)	1.982	1.974	2.028	1.990	2.030	1.933	1.938	1.957	1.962
*N*_O′(fw)_	—	1						2	
Cu–O′_fw_ (Å)	—	2.393	—	—	—	—	—	2.511	—
*N*_T_	1	2	2	1	2	1	1	2	1
Cu–T (Å)	2.782	2.714	2.805	2.690	2.655	2.681	2.707	2.811	2.709

Once DFT has been employed to obtain a set of stable configurations, EXAFS fitting of the spectra collected after O_2_-activation and He-activation provided an effective and computationally inexpensive way to perform a wideband screening of the possible candidate models (see ESI Sec. 1.4.2† for details on the fitting procedure). Subsequently the simulations of XANES and XES were performed for the same structures. The motivation for that was the higher sensitivity of XANES and XES methods to coordination bond angles with respect to EXAFS. Thus, the goal was to discriminate between the DFT structures with similar coordination numbers, but substantially different three-dimensional arrangements of the ligands. It is worth noting that for each structure the whole optimized cluster was taken into account in order to perform XANES and XES calculations (ESI Sec. 1.4.3†), in contrast to EXAFS where only the atoms of the corresponding ring at distances of up to 3.5 Å from Cu were considered.

### 2.4 EXAFS fitting for O_2_-activated and He-activated Cu-SSZ-13

#### 2.4.1 O_2_-activated Cu-SSZ-13: EXAFS screening of different DFT-optimized models for Cu(ii) sites

**Fig. 6:**
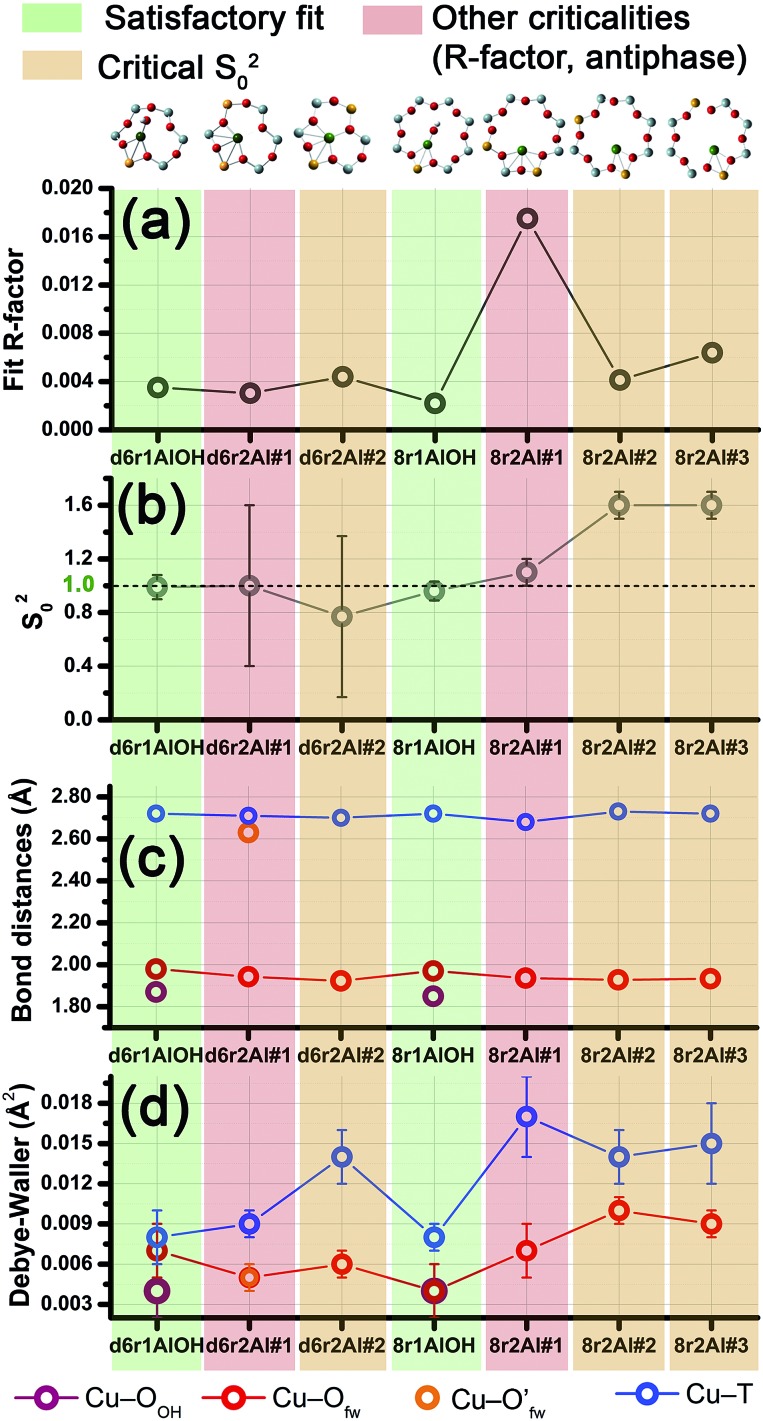
Graphical summary of the results obtained by fitting the experimental EXAFS spectrum collected at 400 °C for O_2_-activated Cu-SSZ-13 on the basis of the different DFT-optimized models for Cu(ii) sites in the d6r and 8r units of the CHA framework. For each model we report: (a) the fit *R*-factor values; (b) the passive amplitude reduction factor *S*_0_^2^ (ideally equal to one, *S*_0_^2^ > 1 suggests underestimated coordination numbers in the model, whereas *S*_0_^2^ < 1 indicates overestimated coordination numbers); (c) the average EXAFS-optimized bond distances and (d) DW factors for each coordination shell included in the fitting model. Vertical stripes of different colours are used to highlight the reliability level of the different fits, with the following colour code: satisfactory fits, green; fits resulting in critical *S*_0_^2^ values, orange; fits showing other criticalities, *e.g.* remarkable increase in *R*-factor value or strong antiphase effects, red.

Fig. 6 summarizes the results obtained by fitting the experimental EXAFS spectrum collected at 400 °C for O_2_-activated Cu-SSZ-13 on the basis of the previously reported DFT-optimized models for Cu(ii) sites in the d6r and 8r units. A full report on the optimized parameters resulting from the seven fits performed can be found in the ESI Sec. 5.† From the graphical summary reported in Fig. 6, we realize that the configurations corresponding to a first-shell coordination number *N*_O(fw)_ = 4 and *N*_O(fw)_ = 2 (mostly associated to distant 2Z^–^ sites in the larger 8r units) resulted in *S*_0_^2^ values of ∼0.8 and ∼1.6, respectively. Conversely, when three-coordinated Cu(ii) sites are considered (bonded to three O_fw_ atoms or to two O_fw_ atoms and to the (OH)^–^ extra-ligand at a slightly shorter distance), *S*_0_^2^ values equal to the optimal value of ∼1 within their errors are observed. Hence, the fit results support the stabilization of 3-coordinated Cu(ii) sites after O_2_-activation, thus ruling out the configurations involving 2- and 4-coordinated Cu centres, at least as dominant structural components under our experimental conditions. This is in agreement with previous EXAFS studies on O_2_-activated Cu-SSZ-13, which reported an average first-shell oxygen coordination number of around three.^3,14^ However, this result was preferentially associated to bare isolated Cu(ii) sites in the d6r (equivalent to our d6r2Al#1, 2 models) whereas, to the best of our knowledge, the OH-like models, which exhibit a very similar first-shell coordination, have never been considered as plausible alternatives for the interpretation of the EXAFS signal of the O_2_-activated catalyst.

**Fig. 7:**
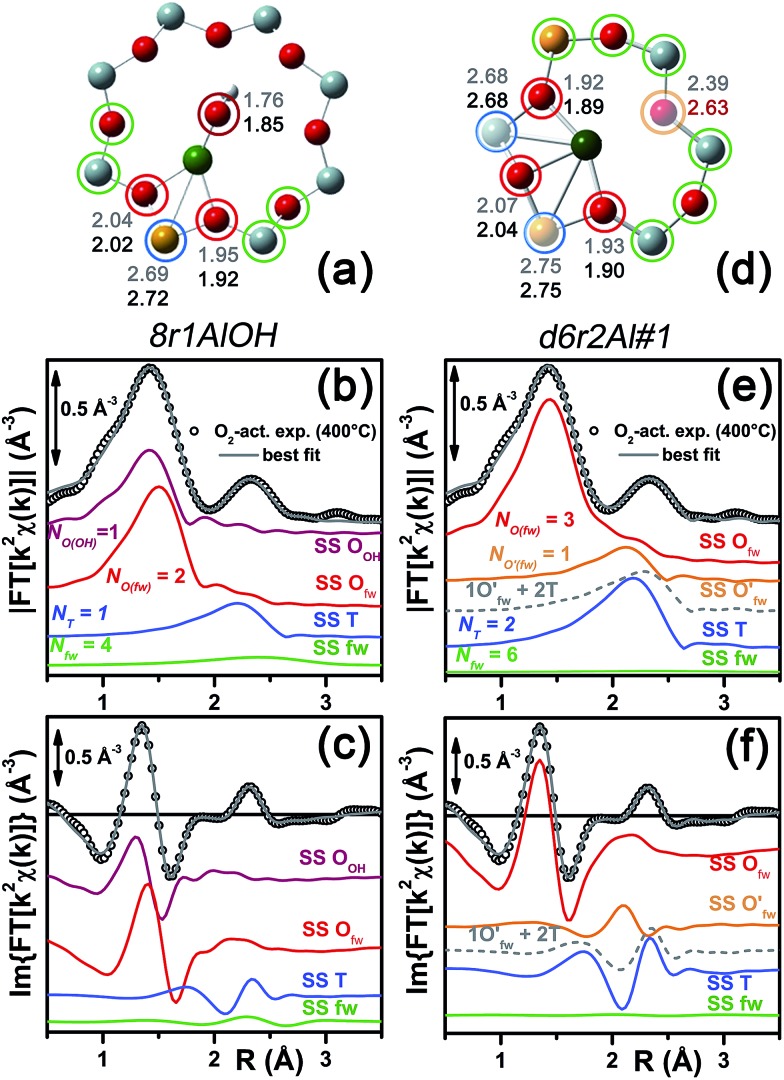
(a and d) Representation of the DFT-optimized (a) 8r1AlOH and (d) d6r2Al#1 geometries; atom colour code: Cu, green; H, white; O, red; Al, gold, Si, gray. Coloured circles highlight the different shells of neighbouring atoms included in the fitting model. Starting DFT-optimized bond distances for each atom from Cu (absorber in our XAS experiment) are reported in grey, while the corresponding values refined by EXAFS fitting are shown in black (distances are indicated in Å). (b and c) Comparison between the experimental EXAFS spectrum of the O_2_-activated Cu-SSZ-13 catalyst and corresponding best fit performed from the 8r1AlOH model; both modulus and imaginary part of the FT are shown in panels (b) and (c) respectively, together with the different SS path contributions to the total signal, with the same colour code employed in part (a) and vertically translated for the sake of clarity. (e and f) The same as panels (b and c) but using the d6r2Al#1 model as an input for the fit. Gray dashed lines indicate the residual signal after summing the O′_fw_ and T contributions. For quantitative values of the parameters optimized in the two fits, see Table 2.

Among the favored 3-coordinated configurations, the 8r2Al#1 model provided an unsatisfactory fit due to a drastic increase of the *R*-factor value (*R* = 0.0180) with respect to the other geometries tested, and also due to a severe damping of the two SS Cu–T contributions by unreliably high DW values. Conversely, the lowest overall *R*-factor was found for the 8r1AlOH model (*R* = 0.0022), evidencing an excellent line-to-line agreement between the experimental and best-fit spectra (see Fig. 7b and c). An almost equivalent *R*-factor was observed considering both the formation of the [CuOH]^+^ complex in the d6r (model d6r1AlOH, *R* = 0.0035) and the stabilization of the cation next to 2Z^–^ sites in the d6r, in the Al–T–Al configuration (model d6r2Al#1, *R* = 0.0030).

The two fits performed on the basis of the extra-ligand-like configurations resulted in very similar values for all of the parameters optimized, reflecting the high similarity of the two local environments around the Cu(ii) sites in both d6r and 8r. Here, only slight deviations from the structural minima found by DFT (Δ*R*_i_ < 0.1 Å) and reliable DW factor values (consistent with high-temperature data collection) were observed. As an example, Table 2 reports the best-fit parameters obtained for the 8r1AlOH case. In addition, in Fig. 7b and c we compare the experimental magnitude and imaginary part of the FT spectrum of the O_2_-activated Cu-SSZ-13 catalyst to the corresponding best-fit curves obtained for the 8r1AlOH model. The individual SS path contributions to the total signal from each included shell of neighbouring atoms are also shown.

**Table 2:** Best-fit parameters optimized by EXAFS fits of the *k* ^2^-weighted spectrum of O_2_-activated Cu-SSZ-13 (data collection at 400 °C), employing as a starting guess two selected DFT-optimized geometries for Cu(ii) sites, *i.e.* the 8r1AlOH and d6r2Al#1 models (a detailed report on the EXAFS fits performed from all the Cu(ii) models tested is reported in ESI Sec. 5†). The fit was performed in *R*-space, in the range (1.0–3.2) Å, employing the *k*-range (2.4–12.4) Å^–1^ for the FT, resulting in a number of independent points *N*_ind_ = 2Δ*k*Δ*R*/π > 14

Best-fit parameters for O_2_-activated Cu-SSZ-13 – Cu(ii) sites		
Optimized parameters	8r1AlOH	d6r2Al#1
*S*_0_^2^	0.96 ± 0.07	1.00 ± 0.06
Δ*E* (eV)	–1.8 ± 0.6	–1.4 ± 0.9
*R*-factor	0.00220	0.00304
*N*_par_	10	9
*R*_OH_ (Å)	1.85 ± 0.02	—
*σ* ^2^_OH_ (Å^2^)	0.004 ± 0.002	—
*N*_OH_	1	—
*R*_O(fw)_ (Å)	1.97 ± 0.01	1.94 ± 0.01
*σ* ^2^_O(fw)_ (Å^2^)	0.004 ± 0.002	0.005 ± 0.001
*N*_O(fw)_	2	3
*R*_O′(fw)_ (Å)	—	2.63 ± 0.01
*σ* ^2^_O′(fw)_ (Å^2^)	—	0.005 ± 0.001
*N*_O′(fw)_	—	1
*R*_T_ (Å)	2.72 ± 0.01	2.71 ± 0.02
*σ* ^2^_T_ (Å^2^)	0.008 ± 0.001	0.009 ± 0.001
*N*_T_	1	2
*α*_fw_	–0.01 ± 0.01	–0.03 ± 0.11
*σ* ^2^_fw_ (Å^2^)	0.022 ± 0.005	0.05 ± 0.5
*N*_fw_	4	6

For the 8r1AlOH model the EXAFS-refined parameters are in good agreement with the DFT-optimized bond distances. In particular, the highest deviation is observed in correspondence to the Cu–O_OH_ bond, refined to 1.85 ± 0.02 Å, with respect to the 1.76 ± 0.02 Å value from DFT analysis. The bond distances between the cation and the framework atoms are only minorly adjusted, with a slight contraction of the O_fw_ shell and a slight elongation of the Cu–Al distance.

The first maximum in the |FT[*k* ^2^ *χ*(*k*)]| spectrum mostly results from the combination of partially overlapping SS paths involving the O_OH_ atom of the extra-ligand group and two O_fw_ atoms. As expected, the structural parameters of the O_OH_ and O_fw_ atoms are affected by rather high mutual correlations. However, test fits performed with three degenerate O_fw_ SS paths, considering an intermediate Cu–O distance of 1.98 Å as a starting guess, resulted in a ∼50% increased *R*-factor value and a doubled σ_O(fw)_^2^ value of ∽(0.010 ± 0.001) Å^2^. Hence, EXAFS is able to distinguish an oxygen ligand located at a significantly shorter distance to Cu with respect to the two O_fw_ atoms.

Notably, we could consider a O^–^ group as an alternative extra-ligand, charge balancing the Cu^2+^ cations in the proximity of 1Z^–^ sites. Unfortunately, discrimination by XAS between (OH)^–^ and O^–^ extra-ligands in the Cu coordination sphere is unfeasible, due to the weak scattering amplitude of H. Nonetheless, by combining XAS and FTIR results we can safely state that for a major fraction of the sites the O_2_-activation results in the formation of [CuOH]^+^ species. Indeed, the presence of (OH)^–^ groups on Cu in the O_2_-activated Cu-SSZ-13 is unequivocally demonstrated by the appearance of the bands at 3656 and 905 cm^–1^, assigned to the *ν*(O–H) and *δ*(O–H) modes in the [CuOH]^+^ complex, respectively. Conversely, the stabilization of [CuO]^+^ monomeric moieties would be incompatible with such vibrational fingerprints.

The second maximum in the experimental |FT[*k* ^2^ *χ*(*k*)]| mainly originates from an individual Cu–Al SS path, with minor contributions on the low-*R* side from the tails of the first-shell Cu–O_fw_ paths, and on the high-*R* side from the SS involving the other O/Si atoms of the 8r unit falling in the considered *R*-range (up to 3.5 Å from the Cu absorber). This assignment quantitatively confirms that the appearance of a well defined coordination shell in the 2.0–2.8 Å range (phase-uncorrected) during activation can be considered as a fingerprint for the increased interaction of the Cu ions with the zeolite framework,^22^ concomitantly to the progressive replacement of the O(H_2_O) ligands with the O_fw_ ones upon thermal dehydration.

It is worth noting that the high-scattering amplitude Cu–Cu SS paths of Cu–O–Cu dimeric species are also expected to fall in this *R*-space region, corresponding to a quite broad distribution of Cu–Cu distances in the 2.73–2.91 Å range, according to the previous literature on Cu–zeolites.^44,63–65^ Nevertheless, the high quality of the EXAFS fits performed without considering any metal–metal scattering path allows the exclusion of a major contribution from such species in the O_2_-activated Cu-SSZ-13 catalyst. A different behavior was observed, *e.g.* in Cu-ZSM-5 and Cu-IM-5 zeolites, with a comparable Cu/Al ratio,^49^ where the second coordination shell of the experimental FT EXAFS spectra was successfully fitted by only including in the refinement both a Cu–Al and a Cu–Cu contribution. It is worth noting that the detection of a minor fraction of Cu(ii) sites (indicatively <10% of the total) occurring as Cu_*x*_O_*y*_ (*x* = 2, *y* ≥ 1) species is beyond the sensitivity limit of the EXAFS technique, especially if flexible dimers with highly dispersed Cu–Cu bond distances are concerned. However, the occurrence of such species as a dominant structural component (indicatively >50% of the total Cu sites probed by XAS) in activated Cu-SSZ-13 is incompatible with our XAS results, even under the assumption of a high dispersion. Indeed, if a high concentration of these species had been present, their structural flexibility would have resulted in dampened and broadened, but still detectable, Cu–Cu SS contributions.

On this basis, the Cu–O–Cu dimers nestled in the 8r cages, recently claimed by Verma *et al.* ^19^ to be the active sites for dry NO oxidation on Cu-SSZ-13 above a Cu/Al atomic ratio = 0.2, could occur only as a minor fraction of the total Cu sites in our Cu-SSZ-13 sample (Cu/Al = 0.44). In addition, preliminary XAS measurements on an O_2_-activated low Cu-loading Cu-SSZ-13 sample (Cu/Al ∼0.13) clearly show EXAFS features substantially equivalent to those reported here for the high-loading material (see ESI Sec. 6†). In particular, the EXAFS spectrum for the O_2_-activated sample with Cu/Al ∼0.13 always shows a well-defined coordination shell in the 2.0–2.8 Å range (phase-uncorrected), with comparable magnitude and only slightly shifted to higher distances. This evidence further discourages the possibility of a significant contribution by Cu–Cu SS paths from Cu–O–Cu dimers to such an EXAFS feature in Cu-SSZ-13 catalysts even with high Cu-loading.

In summary, the 8r1AlOH model provided a very good reproduction of the experimental spectrum collected for O_2_-activated Cu-SSZ-13, with reliable values of all the physical and structural parameters refined and only minor deviations from the DFT-optimized geometry employed as starting guesses in the fit. Equivalently good fits were obtained for the d6r1AlOH model (see ESI Sec. 5, Table S2†). Thus, in these conditions the 8r1AlOH and dr1AlOH models can be hardly distinguished by EXAFS. However, it is worth noting that the Cu–T distance is refined to the same value of 2.72 Å starting from both DFT geometries, which predict Cu–T distances of 2.69 and 2.78 Å in 8r and d6r, respectively. This suggests that the [Cu^2+^(OH)^–^]^+^ complexes are preferentially stabilized in the proximity of 1Z^–^ sites in the plane of the larger 8r units of the SSZ-13 framework, as was also very recently confirmed by Andersen *et al.* ^16^ by Rietveld/MEM analysis of synchrotron powder X-ray diffraction data.

Apart from the two OH-like configurations, a very good line-to-line agreement between experimental and best-fit lines was also obtained for the d6r2Al#1 model, apparently without the occurrence of critical/unphysical values for the refined parameters (see Table 2 and Fig. 6). Consistently, such a coordination environment for isolated Cu(ii) sites in the d6r has been proposed as the dominant one in previous EXAFS works,^3,14^ also based on its superior stability from DFT analysis. The experimental FT spectra of the O_2_-activated Cu-SSZ-13 and corresponding best fit curves obtained for the d6r2Al#1 model are also reported in Fig. 7e and f together with the individual SS path contributions from each shell of neighbouring atoms. Here, the first shell is successfully fitted by the SS contribution of three O_fw_ atoms refined at an average distance of (1.94 ± 0.01) Å by a slight contraction from the DFT values (Δ*R*_O(fw)_ = –0.03 Å), with a DW factor *σ* ^2^_O(fw)_ = (0.005 ± 0.001) Å^2^, slightly increased with respect to the 8r1AlOH case. However, the second coordination shell is reproduced only by a dramatic elongation of the Cu–O′_fw_ distance up to (2.63 ± 0.01) Å (Δ*R*_O′(fw)_ = +0.24 Å) with respect to the stable DFT configuration. Such a high distortion results in the almost perfect antiphase between the SS path involving the elongated O′_fw_ and that involving the two T atoms, which on the contrary are left exactly in their original DFT positions. The effective signal which results from the partial cancellation of the two contributions is shown in Fig. 7e and f as a gray dashed line, and it is essentially equivalent to the SS path involving the individual Al atom in the 8r1AlOH configuration (see parts b and c in the same figure). Notably, suspiciously high correlations are also observed between the structural parameters of the O′_fw_ and T shells (Δ*R*_O′(fw)_ & Δ*R*_T_ → 0.95). The destructive interference between EXAFS paths cannot be considered *a priori* as critical, and a few cases have been reported in which these severe “cancellation” effects are a distinguishing feature of the EXAFS signature for the studied material.^66–68^ However this is likely not the case, due to the drastic distortion of the d6r unit with respect to the DFT-optimized geometry and the similarity of the resulting signal in the 2.0–2.8 Å range to that resulting from an individual Al atom located at 2.72 Å from Cu, as in the 8r1AlOH and d6r1AlOH models. In addition, test fits performed by modeling the Cu–O′_fw_ SS path with an independent DW factor resulted in unphysically high values for the latter parameter. Even more drastically, by simply removing this SS path from the fit model, the fit was satisfactory, but with remarkably increased DW factors for the T shell (up to 0.016 Å^2^) with respect to the extra-ligand-like configurations, probably to adjust the presence of two T atoms instead of one in the coordination shell of interest.

Based on these observations, our analysis strongly discourages the stabilization of a major fraction of Cu(ii) sites next to 2Z^–^ sites in the d6r according to the d6r2Al#1 model after O_2_-activation. If present, this configuration should involve a limited percentage of Cu sites, so that their contribution in the EXAFS spectrum could not be resolved within our data quality (a fraction of 9% for the d6r2Al#1 site was recently suggested from EPR^18^ performed on a similar Cu-SSZ-13 sample, but using slightly different dehydration time/temperature conditions). Conversely, the formation of [Cu^2+^(OH)^–^]^+^ complexes in 8r units next to 1Z^–^ sites ensures the best compatibility with our experimental spectrum and should be assumed as the most representative model for the average structural environment of Cu(ii) sites in O_2_-activated Cu-SSZ-13.

#### 2.4.2 EXAFS analysis of Cu(i) sites in the d6r and 8r units

**Fig. 8:**
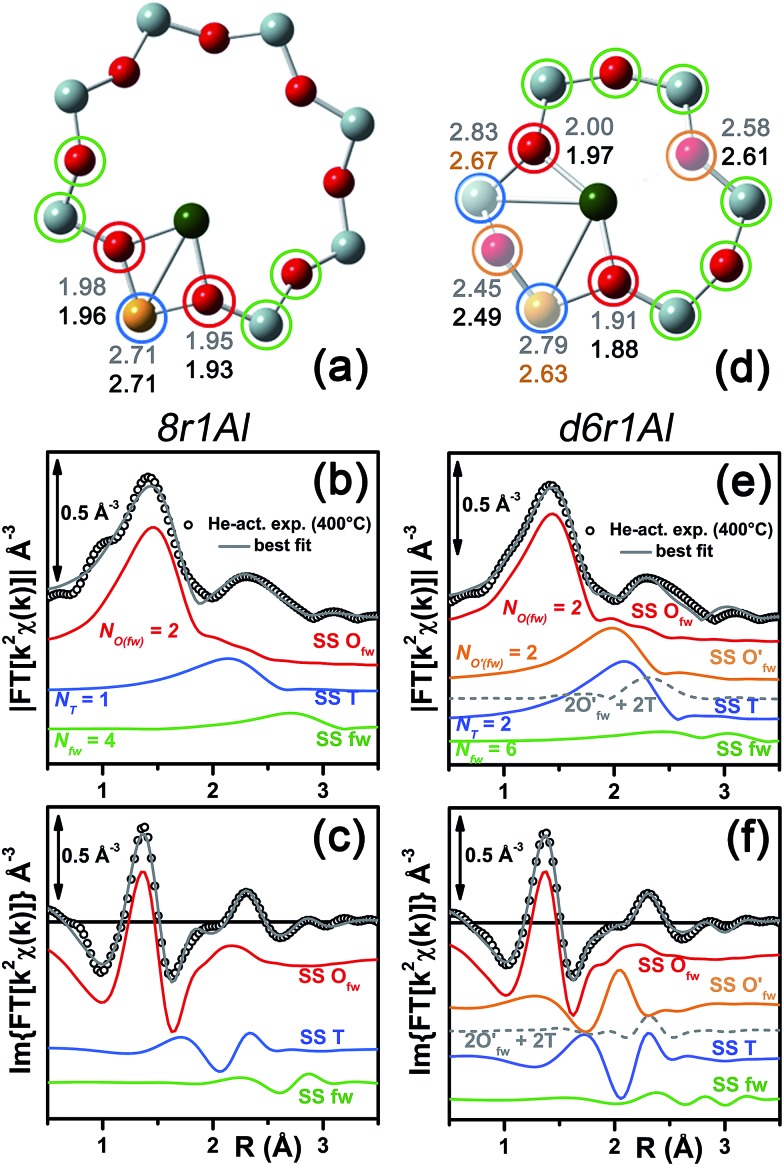
(a and d) Representation of the DFT-optimized (a) 8r1Al and (d) d6r1Al geometries; atom colour code: Cu, green; O, red; Al, gold, Si, gray. Coloured circles highlight the different shells of neighbouring atoms included in the fitting model. Starting DFT-optimized bond distances for each atom from Cu (absorber in our XAS experiment) are reported in grey, while the corresponding values refined by EXAFS fitting are shown in black (distances are indicated in Å). (b and c) Comparison between the experimental EXAFS spectrum of the He-activated Cu-SSZ-13 catalyst and the corresponding best-fit performed from the 8r1Al model; both modulus and imaginary part of the FT are shown in panel (b) and (c) respectively, together with the different SS path contributions to the total signal, with the same colour code employed in part (a) and vertically translated for the sake of clarity. (f and g) The same as parts (b and c) but using the d6r1Al model as an input for the fit. Gray dashed lines indicate the residual signal after summing the O′_fw_ and T contributions. For quantitative values of the parameters optimized in the two fits, see Table 3.

We tested the compatibility of the DFT-optimized models for “bare” Cu(i) sites in the proximity of 1Z^–^ sites in both d6r and 8r by fitting the EXAFS spectrum collected on the He-activated catalyst. These configurations could be reached upon the progressive release of the extra-ligand group and subsequent readjustments of the reduced cation in its framework positions, as supported by the evolution of the XAS features during He-activation (see Fig. 4). Table 3 reports the respective best-fit parameters obtained on the basis of the 8r1Al and d6r1Al models, while the corresponding best-fit curves are reported in Fig. 8. Both the models were found to be compatible with the experimental spectrum, with *S*_0_^2^ values equal to units within their fit errors. The overall fit quality was, however, lower with respect to the O_2_-activated Cu-SSZ-13 case, as reflected by the higher, although still satisfactory, *R*-factor values.

**Table 3:** Best-fit parameters optimized by EXAFS fits of the *k* ^2^-weighted spectrum of He-activated Cu-SSZ-13 (data collection at 400 °C), employing as starting guesses DFT-optimized geometries for Cu(i) sites in the 8r and d6r units of the zeolite framework, *i.e.* the 8r1Al and d6r1Al models. The fit was performed in *R*-space, in the range 1.0–3.2 Å, employing the *k*-range 2.4–12.4 Å^–1^ for the FT, resulting in a number of independent points *N*_ind_ = 2Δ*k*Δ*R*/π > 14

Best-fit parameters for He-activated Cu-SSZ-13 – Cu(i) sites		
Optimized parameters	8r1Al	d6r1Al
*S*_0_^2^	1.1 ± 0.1	0.9 ± 0.1
Δ*E* (eV)	–3 ± 1	–5 ± 1
*R*-factor	0.01510	0.01073
*N*_par_	8	9
*R*_O(fw)_ (Å)	1.94 ± 0.01	1.92 ± 0.01
*σ* ^2^_O(fw)_ (Å^2^)	0.011 ± 0.002	0.006 ± 0.002
*N*_O(fw)_	2	2
*R*_O′(fw)_ (Å)	—	2.55 ± 0.05
*σ* ^2^_O′(fw)_ (Å^2^)	—	0.006 ± 0.002
*N*_O′(fw)_	—	2
*R*_T_ (Å)	2.71 ± 0.02	2.65 ± 0.04
*σ* ^2^_T_ (Å^2^)	0.015	0.012 ± 0.002
*N*_T_	1	2
*α*_fw_	0.00 ± 0.01	0.01 ± 0.01
*σ* ^2^_fw_ (Å^2^)	0.008 ± 0.003	0.008 ± 0.003
*N*_fw_	4	6

In particular, starting with the 8r1Al model, the two coordination shells observed in the experimental spectrum were well reproduced employing only two Cu–O_fw_ and one Cu–T SS paths, with additional contribution in the high-*R* region from the remaining atoms in the 8r. The corresponding bond distances from the cation are only slightly varied with respect to the DFT-optimized geometry, and adjusted to ∼1.94 Å and ∼2.71 Å for the O_fw_ and T coordination shells, respectively. However, as it can be noted from Table 3, the DW parameters for the two sets of paths were optimized to higher values with respect to the O_2_-activated Cu-SSZ-13 case. These are still physically meaningful values, and the paths contribution to the two maxima of the experimental |FT[*k* ^2^ *χ*(*k*)]| curve can be clearly identified (see Fig. 8b and c). Nevertheless, the higher DW values observed are clearly symptomatic of an increased structural disorder in the local environment of Cu(i) sites formed upon He-activation, whereas the thermal contribution to DW factors is unvaried with respect to the O_2_-activated case, having collected the two spectra at the same temperature of 400 °C.

Further insights can be obtained by analyzing the results of the EXAFS fit performed according to the d6r1Al model. Here, the presence of an additional parameter in the fit model (radial shift of the two additional O′_fw_ atoms at the average Cu–O′_fw_ distance ∼2.51 Å from DFT, see Table 1) ensured a moderate decrease in the *R*-factor value. A reduction of the DW values for all of the included coordination shells was also observed with respect to the fit performed using the 8r1Al model, although DW factors still significantly higher with respect to the O_2_-activated material were found. Nevertheless, the best-fit is obtained in correspondence to a pronounced contraction of the Cu–T distance, with Δ*R*_T_ ∼ –0.16 Å from the DFT values. This distortion is less critical with respect to what was obtained using the d6r2Al#1 model to fit the spectrum of the O_2_-activated sample (Δ*R*_O′(fw)_ ∼ +0.24 Å) but severe enough to suggest a partial inability of this model to reproduce by itself the experimental spectrum.

In addition, the radial shift parameters for the *R*_O′(fw)_ and T coordination shells are affected by a very high mutual correlation: Δ*R*_O′(fw)_ & Δ*R*_T_ → 0.96. As can be observed in Fig. 8f, the respective SS paths are adjusted to almost perfect antiphase, even though in this case the residual signal slightly differs from the Cu–T SS path refined for the 8r1Al model. Hence, the fit routine is not just trying to restore the local environment of the 8r1Al model, but likely bringing up a secondary contribution from the d6r1Al geometry. Not surprisingly, it was impossible to refine two independent structural components from both models, due to the limited “structural contrast” between the Cu(i) local geometries in 8r and d6r, resulting in correlations >0.9 between all the structural parameters involved. Finally, it is worth noting that the d6r1Al model closely resembles the d6r2Al#1 Cu(ii) configuration in terms of Cu nearest neighbours, upon replacement of one out of two Al atoms by Si in the T-site of the d6r ring (indistinguishable by EXAFS). Hence, the secondary contribution overlapped to the major 8r1Al component could also be assigned to difficultly reducible Cu(ii) sites in the d6r. This possibility is corroborated by the already mentioned work by Godiksen *et al.*,^18^ where the residual EPR signal from Cu-SSZ-13 dehydrated at 250 °C in a He-flow has been related to the persistence of two well-defined EPR-active Cu(ii) sites in the d6r, each accounting for ∼9% of the total Cu.

In conclusion, EXAFS analysis of He-activated Cu-SSZ-13 indicates the 8r1Al model as the most reliable average configuration. However, the fitting results also pointed out a higher structural disorder with respect to what was observed for the O_2_-activated sample, in concomitance to the probable presence of an additional minor contribution, either from “bare” Cu(i) sites in d6r, which arrange in a non-equivalent coordination environment, or from residual, difficultly reducible Cu(ii) species whose presence is confirmed by HERFD XANES (see below).

### 2.5 DFT-assisted XANES and XES simulations for O_2_-activated and He-activated Cu-SSZ-13

Along with the EXAFS fitting, calculations of the XANES and XES spectra for all of the obtained DFT models were performed in order to complement the analysis. Since the features of the XANES spectra, recorded using a conventional transmission setup, are rather broad and not always clearly distinguishable, we will use HERFD XANES data with much more pronounced peaks for the comparison with theory. These spectra were collected in sample environment conditions analogous to the corresponding conventional XANES and therefore are fully equivalent to them.

**Fig. 9:**
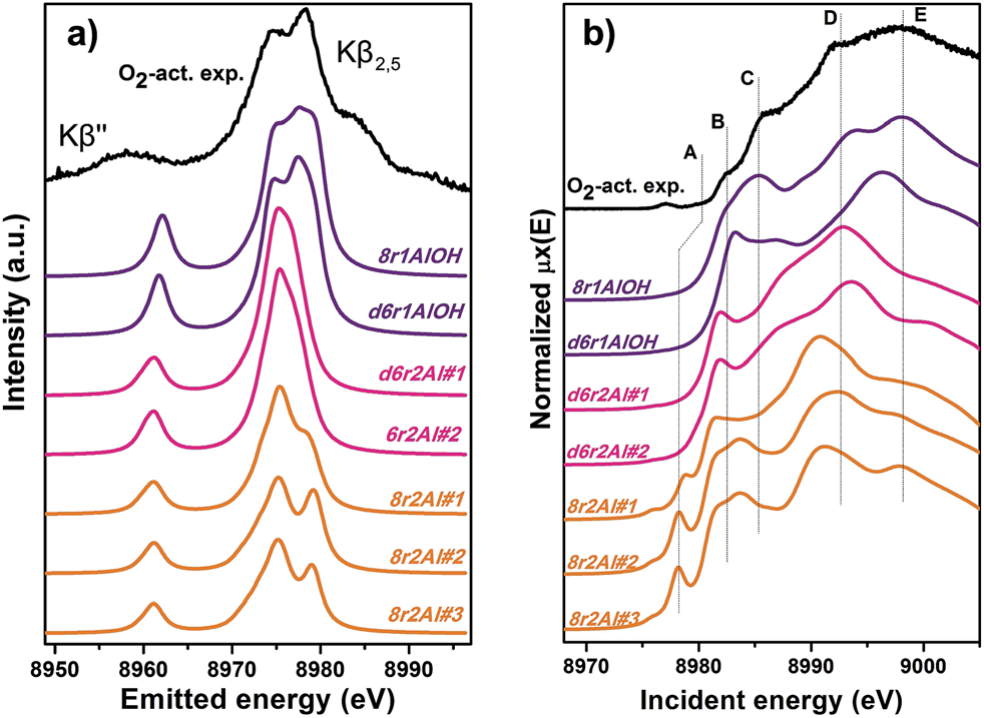
Experimental XES (a) and HERFD XANES (b) spectra of O_2_-activated Cu-SSZ-13 together with the simulated curves for different DFT models for Cu(ii) sites presented in Fig. 5. Spectra are shifted along the vertical axis for the sake of clarity.

Fig. 9a shows the results of the XES simulations for the trial structures of Cu(ii). Simulated spectra are divided into three groups: those that contain the extra-ligand OH group (purple), those with 2 Al atoms in the d6r (pink) and finally those with 2 Al atoms in the 8r (orange). Similarly to EXAFS, the XES simulations support the OH-like structures, since only in these cases it is possible to reproduce the double-peaked Kβ_2,5_ line as it is present in the experiment. An interesting observation is that all of the simulations fail to reproduce correctly the separation between Kβ_2,5_ and Kβ′′ emission lines. A similar underestimation of this distance in the calculated XES spectra of various Cu model compounds was reported recently by Vegelius *et al.* ^69^ This might indicate a systematic error in the treatment of ligand 2s orbitals by the chosen theoretical approaches, since that is the main origin of Kβ′′ satellite intensity.

Each of the three groups of XANES spectra (Fig. 9b) has its own characteristic features. In particular, all 8r2Al structures (orange lines) exhibit a distinct peak in the lower energy region (feature A), that is completely absent in the other simulated spectra. In the experimental data, one can only observe a very minor contribution from this transition. Along with the general mismatch in the shape of the spectra, this allows us to rule out these low-coordinated configurations as the major species for O_2_-activated Cu-SSZ-13. Disagreement with the experiment in the shape of the main maxima (peaks D and E) does not favor structures with 2 Al atoms in the d6r (pink curves) as well. The best results in terms of peak positions were obtained for the 8r1AlOH model, in agreement with the EXAFS fitting results reported above. Notably, the spectrum of the d6r1AlOH structure provided a significantly worse reproduction of the experimental curve, demonstrating a higher sensitivity of XANES compared to EXAFS and XES, where the two OH-like models in d6r and 8r yielded very similar results.

**Fig. 10:**
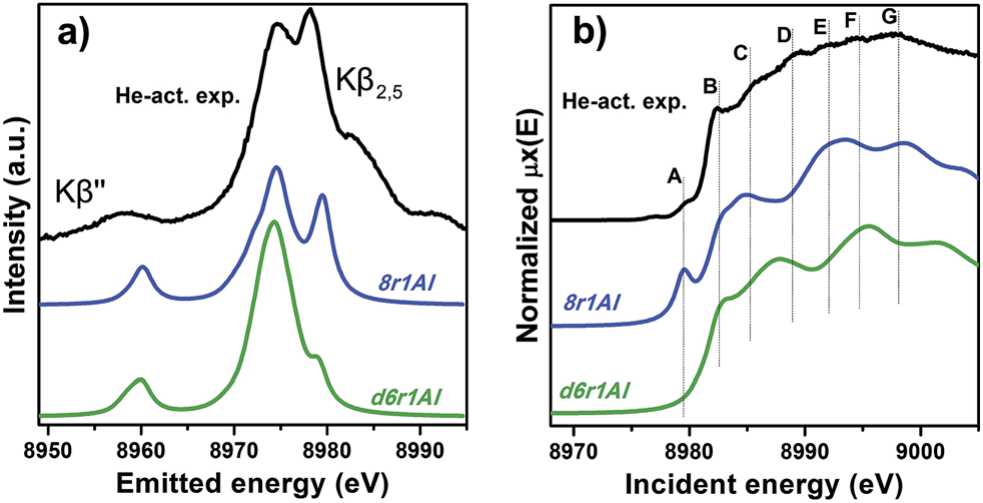
Experimental XES (a) and HERFD XANES (b) spectra of He-activated Cu-SSZ-13 together with the simulated curves for different DFT models for Cu(i) sites presented in Fig. 5. Spectra are shifted along the vertical axis for the sake of clarity.

However, it is worth noting that for a relatively inhomogeneous system like Cu-SSZ-13 the enhanced sensitivity of XANES can also be a disadvantage, since all of the Cu species present in the sample contribute in a different way to the signal. Hence, it is not surprising that none of the simulations are in line-to-line agreement with the experiment. This problem should have been faced by Deka *et al.* ^31^ as well, who have reported to the best of our knowledge the only Cu K-edge XANES simulation of the activated Cu-SSZ-13 up to now. Nonetheless, the simulation has allowed them to demonstrate that copper shifts towards the framework rather than staying in the middle of the cavity. The problem of inhomogeneity is even more evident looking at the XES and XANES simulations of the spectra collected for the He-activated catalyst (Fig. 10). The conclusion drawn from the EXAFS data, that there is a much higher degree of disorder in the local environment of copper, is confirmed by rather strong discrepancies between experiment and theory. Nonetheless, XES simulations (Fig. 10a) suggest that the 8r structure is more favorable, since it yields the double-peaked Kβ_2,5_, unlike the competing d6r model. The large number of peaks present in the experimental HERFD XANES spectra suggests that several non-negligible species are present in the sample at these activation conditions, in agreement with the EPR results of Godiksen *et al.* ^18^ Interestingly, peaks in the calculated spectra of the two trial structures are often in antiphase, which might explain a broad main maximum with several weak oscillations (features D–G), visible in the experiment. It indicates that both of these two species are likely to exist in the material, 8r1Al being more abundant since only this structure can be the origin of the pre-edge peak A, whose intensity in the experiment increased significantly compared to the case of O_2_-activation. Remarkably, the 1s → 3d peak is still present in the experimental data, suggesting a residual contribution of oxidized Cu(ii) species as well. However, their amount should be rather low, since the peak is so weak that it is visible only in the high-resolution mode due to a very low background, and completely absent in the conventional XANES spectrum.

In summary, it is possible to conclude that the XES and XANES data support the findings of DFT and EXAFS. The performed simulations suggest that copper is more likely to dwell in the larger 8-membered ring cavities than in smaller 6-membered rings. Likewise, the formation of [CuOH]^+^ complexes is favored in the case of the activation in O_2_ compared to the alternative of “bare” Cu^2+^ ions in the rings that contain two aluminum atoms. Despite the fact that the aforementioned presence of many rather different Cu species in the material worsens the agreement of the simulated XES and XANES spectra with the experimental, these methods proved to be an invaluable complement for the EXAFS and DFT data, which is particularly important when studying such complex systems like Cu-SSZ-13.

## 3. Conclusions and perspectives

**Scheme 1:**
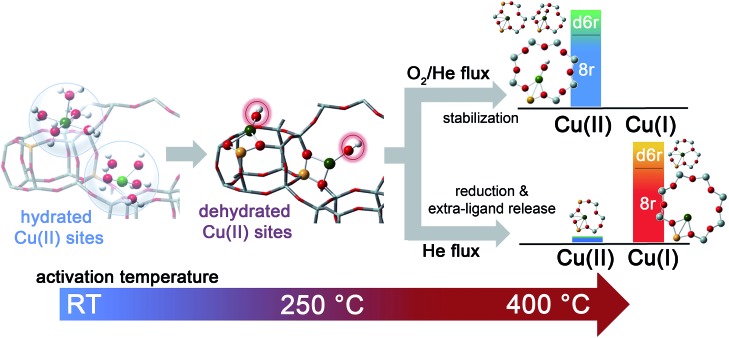
Schematic representation of Cu-speciation in the Cu-SSZ-13 catalyst as a function of the dehydration temperature and conditions. In the O_2_-activated Cu-SSZ-13 we found virtually no traces of Cu(i) within the experimental incertitude of the employed techniques. Conversely, the presence of a minor fraction (around 10%) of Cu(ii) in the He-activated sample was detectable in HERFD XANES spectra. The larger geometries depicted in the right side of the scheme are the dominant structural components identified by XAS, XES and FTIR in the Cu-SSZ-13 sample investigated in the present work.

The dehydration process in the Cu-SSZ-13 catalyst has been monitored by XAS and FTIR spectroscopy in the RT–400 °C temperature range. Depending on the adopted thermal treatments, namely O_2_-activation and He-activation, different Cu species have been found to be formed. Data analysis revealed that the dehydration process of Cu cations is substantially completed at 250 °C, with the formation of dehydrated [CuOH]^+^ species strongly interacting with the zeolite framework. These species are maintained even at higher temperatures only if a certain amount of O_2_ is present in the gas feed. Otherwise, they undergo virtually total “self-reduction” as a consequence of an OH extra-ligand loss. The reversibility of the process has been verified at 400 °C, demonstrating the high Cu(ii)/Cu(i) redox capability. On the basis of these novel findings, the dehydration process and the effect of different activation conditions on the Cu-speciation in the Cu-SSZ-13 catalyst can be revised according to Scheme 1.

We tested by detailed EXAFS analysis in combination with XANES/XES simulations different DFT-optimized structures, and by comparison with the experimental data we found that the majority of dehydrated Cu species are hosted in close proximity to 1-Al sites, preferentially in 8r units of the SSZ-13 matrix. In particular, our results strongly support the tri-coordinated [CuOH]^+^ species as the dominant structural component upon O_2_-activation; conversely, bi-coordinated bare Cu^+^ cations in 8r units have been found to represent the most abundant configuration upon He-activation. Other configurations, if present, are supposed to occur in percentages small enough to hamper the deconvolution of their contributions from the total XAS/XES signals, inherently averaged over all of the Cu sites. Nonetheless, the formation of a significant fraction of dimeric oxo-Cu species, *i.e.* Cu_*x*_O_*y*_ (per mole Cu, *x* ≥ 2, *y* ≥ 1), seems to be very unlikely. This reinforces the idea that the Cu-SSZ-13 catalyst mainly contains monomeric Cu species, in the form of redox-active [CuOH]^+^ complexes, which are thought to play a key role in the NH_3_-SCR mechanism. The present findings pave the way to the future understanding of the nature of the catalytic centres under operating conditions. On one hand, they provide the starting structures that reactants will find when fed onto the activated catalyst. On the other hand, the high stability of both Cu^2+^ and Cu^+^ species identified in the present work as dominant structural components upon O_2_-activation and He-activation, namely [CuOH]^+^ and bare Cu^+^ cations, strongly suggests that these moieties act as key intermediates along the SCR catalytic cycle. Future experiments in *operando* conditions are, however, required to confirm this hypothesis and to unravel the other species involved in the reaction mechanism.
