# Supplementary material for: Revisiting the nature of Cu sites in the activated Cu-SSZ-13 catalyst for SCR reaction
Source: Chem Sci. 2014 Oct 13;6(1):548–63. doi: 10.1039/c4sc02907k (PMC5588737; doi:10.1039/c4sc02907k)
Supplement: Supplementary file 1 [file SC-006-C4SC02907K-s001.pdf]

Electronic Supplementary Material (ESI) for Chemical Science.
This journal is © The Royal Society of Chemistry 2014

Supporting information

Revisiting the nature of Cu-sites in activated Cu-SSZ-13 catalyst for SCR reaction

E. Borfecchia,[a,b] K. A. Lomachenko,[a,e] F. Giordanino,[a,b] H. Falsig,[c] P. Beato,[c]
A. V. Soldatov,[e] S. Bordiga,[a,b] C. Lamberti,* [a,d,e]

[a] Department of Chemistry and INSTM Reference Center, University of Turin, via P. Giuria 7, 10125 Turin, Italy
[b] NIS Centre of Excellence, University of Turin, Italy
[c] Haldor Topsøe A/S, Nymøllevej 55, 2800 Kgs. Lyngby, Denmark
[d] CrisDI center of crystallography, University of Turin, Italy
[e] Southern Federal University, Zorge street 5, 344090 Rostov-on-Don, Russia

Contents

1 Experimental Section 2
1.1 Sample description 2
1.2 In situ FTIR spectroscopy 2
1.3 Synchrotron characterization 2
1.3.1 In situ gas flow setup 2
1.3.2 XAS and XES data collection and reduction procedures 3
1.4 DFT-based analysis of XAS and XES data 3
1.4.1 Computational details 3
1.4.2 EXAFS fitting procedure 4
1.4.3 XANES and XES simulations 5
2 XAS of hydrated Cu-SSZ-13 6
3 Reversibility of the Cu(I) ↔ Cu(II) redox chemistry and extra-ligand loss process upon high-temperature gas-flow switching 7
4 Full report on DFT-optimized geometries 8
5 Full report on EXAFS fitting results on O_2-activated Cu-SSZ-13 – Cu(II) sites 10
6 EXAFS spectra for a low Cu-loading Cu-SSZ-13 sample after O_2-activation @400 °C 12
7 References 12

1 Experimental Section

1.1 Sample description

The Cu-SSZ-13 sample we used in this work is the same we synthesized and characterized in our previous reports [1, 2]. From ICP-OES elemental analysis, it is characterized by Cu/Al = 0.444 and by Si/Al = 13.1.

1.2 In situ FTIR spectroscopy

Powdered sample (around 15 mg) was pelletized, shaped in a self-supported wafer and placed inside a commercial FTIR reactor cell (AABSPEC, #2000-A multimode) which allows to record infrared spectra in a wide range of temperature under flow conditions. Sample was activated at 400 °C for 30 min (heating ramp 5 °C/min) by flowing 30 ml/min of two different gases: i) 50% O_2/He gas mixture; ii) pure helium. Dehydration process has been monitored stepwise by collecting spectra every 5 °C in the whole 30 – 400 °C temperature range. Spectra have been recorded in transmission mode with a resolution of 2 cm^{-1} (number of scans equal to 32) on a Perkin Elmer System 2000 infrared spectrophotometer equipped with a MCT detector.

1.3 Synchrotron characterization

1.3.1 In situ gas flow setup

Fig. S1 shows the employed flow setup (b), and its implementation in combination with the Microtomo reactor cell (a) at the BM23 beamline of the European Synchrotron Radiation Facility (ESRF, Grenoble, France). An equivalent experimental setup was used for HERFD XANES and XES experiments at the ID26 beamline of the ESRF. The gas-flow setup consists of the two main channels, each of them connected with stainless tubes to gas bottles with different composition; the mass flow controller system allows to set the total flow rate and the composition of the reactor feed. For sake of security, each flow channel could be easily switched to venting by means of the dedicated valves. The inert stream could be forced to pass through a water saturator, allowing to reach a certain concentration of water in the final feed. In order to avoid water condensation, part of the line tubes is maintained at 150 °C by means of heater tapes. If needed, the Microtomo reactor volume could be also evacuated at pressure lower than 10^{-4} mbar by means of a turbomolecular pump.

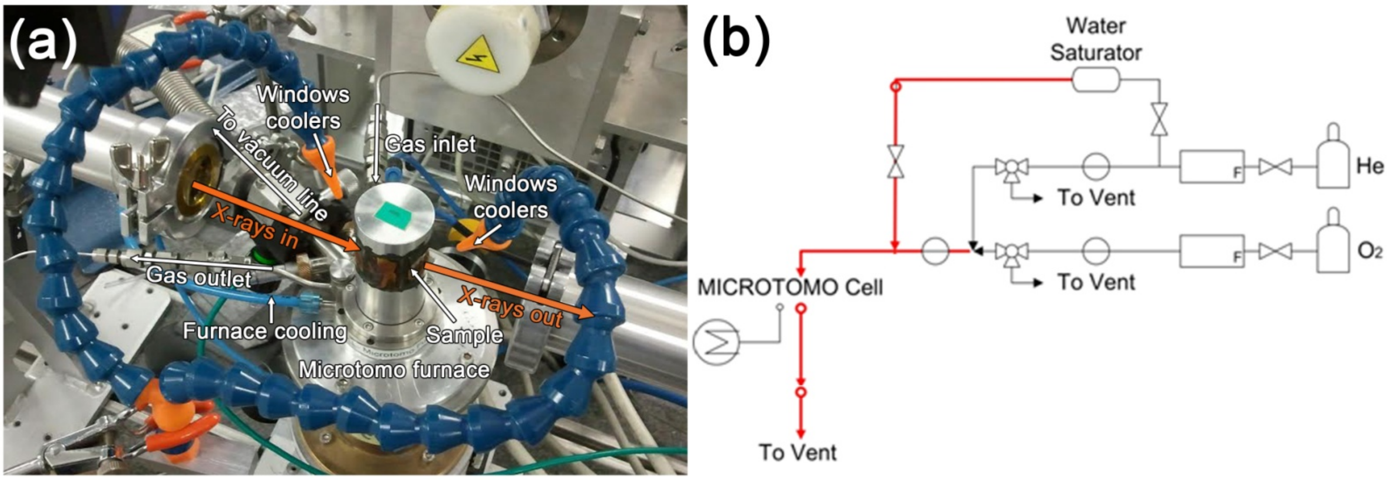

Fig. S1. (a) Picture detail of the Microtmo reactor cell assembled for in situ XAS measurements in flow conditions. (b) Scheme of the whole setup. Red arrows refer to heated tubes in order to avoid water condensation.

The XAS/XES experiments aiming to monitor the activation process of the catalysis and to investigate the final electronic and structural features of Cu-sites in the activated material were performed in conditions comparable to that employed for FTIR characterization. In particular, the Cu-SSZ-13 sample was activated at 400 °C (heating ramp 5 °C/min), and maintained at fixed temperature until a complete stabilization of the XANES features was observed. The activation was performed by flowing in the Microtomo reactor cell 100 ml/min of two different gases: i) 50% O_2/He gas mixture (*O_2-activation*); ii) pure helium ("*He-activation*"). The activation process has been monitored stepwise by XAS collecting spectra every ~ 60 °C in the whole RT – 400 °C temperature range, whereas the hydrated material (at room temperature, RT) and the final states reached after activation (at 400 °C) were characterized by high-quality XAS and XES scans.

It is worth to note that, although all the *in situ* experiments reported hereinafter has been performed using research grade purity gases, it is impossible to exclude the presence of traces of water (in the ppm range) in the gas flow, also considering special affinity of Cu-zeolites towards water. With this respect, high-temperature data collection was preferred for the final states reached by the catalyst after activation, in order to avoid any possibly rehydration of the Cu sites. Nonetheless, water is always present in SCR conditions, and the possible presence of traces of water is in line with a realistic study of the working catalyst.

1.3.2 XAS and XES data collection and reduction procedures

In situ Cu K-edge XAS measurements were carried out at the BM23 beamline of the European Synchrotron Radiation Facility. Data were collected in transmission mode, using double-crystal Si (111) monochromator for the incident energy scan and ionization chambers for the detection of incident and transmitted photons. The chambers were filled with a He/Ar mixture up to 2.2 bar with the partial pressure of argon of 0.1 and 0.3 bar for I_0 and I_1 chambers respectively. For an accurate edge energy calibration, a copper foil was measured simultaneously with all the acquired spectra using a third ionization chamber I_2.[3, 4]. The Cu-SSZ-13 sample was prepared in the form of a self-supporting wafer (~ 100 mg) and fixed inside the Microtomo reactor cell shown in Fig. S1a.

Two different XAS acquisition modes were employed. In particular, we monitored the evolution of the XANES and EXAFS features during activations ramps with faster acquisitions of ~ 13 min, allowing to appreciate the variation of the XAS signal as a function of the temperature (pre-edge region energy step = 5 eV, edge region energy step = 0.3 eV; the EXAFS part of the spectra was collected with variable sampling step in energy, resulting in $\Delta k = 0.05$ Å^{-1}, up to 13 Å^{-1}; integration time was 1 s/point in all regions). The static states of the catalyst reached after full stabilization at fixed temperature (in the hydrated condition and after O_2-activation and He-activation) were characterized by two consecutive higher-quality XAS scans of ~ 30 min, collected up to 13 Å^{-1} with enhanced k-space sampling ($\Delta k = 0.035$ Å^{-1}) and acquisition time in the EXAFS region, that quadratically increased with k from 1 to 4 s/point to account for the low signal-to-noise ratio at high k values). The final spectra employed for quantitative structural refinement are then obtained as the average of the two $\mu(E)$ curves corresponding to the consecutive scans, after checking reproducibility among the two acquisitions.

All the acquired XAS spectra were aligned employing the corresponding Cu metal foil spectra detected by the I_2 ionization chamber and normalized to unity edge jump using the Athena software from the Demeter package.[5] The extraction of the $\chi(k)$ function was also performed using Athena program[5], and R-space EXAFS spectra were obtained by calculating the Fourier transform of the $k^2\chi(k)$ functions in the (2.4 – 12.4) Å^{-1} k-range.

Cu K-edge HERFD XANES and Kβ XES measurements were carried out in ESRF at the ID26 beamline. HERFD XANES spectra were collected in fluorescence mode detecting only photons whose energy corresponded to the maximum intensity of the Cu $K\beta_{1,3}$ emission line (roughly 8906 eV). This selection was made using (800) reflection of one (for O_2-activation experiment) or two (for He-activation experiment) Ge (100) analyzer crystals set up in vertical Rowland geometry. The crystals were spherically bent according to the Johann scheme to focus the fluorescence radiation on the APD detector. For the incident beam a flat double-crystal Si (311) monochromator was used. To measure XES the incident energy was set to 9050 eV, while scans over the emitted energy were performed using the same analyzer crystals as for HERFD XANES. Integration time for each of the emission spectra was around 3 hours, whereas the HERFD XANES of O_2-activated and He-activated samples were the average of 15 and 7 1-minute scans, respectively.

Regarding the experimental data reduction, HERFD XANES were treated in the same way as the conventional ones, but for the sake of comparison with calculations in the end normalization to the main maximum intensity was performed. Valence XES data were scaled to the intensity of $K\beta_{1,3}$ main line and then underwent the background subtraction procedure. Contribution of the $K\beta_{1,3}$ was fitted with four Voigt curves and subtracted, leaving the background-free $K\beta_{2,5}$ and Kβ" features.

1.4 DFT-based analysis of XAS and XES data

1.4.1 Computational details

Structures used for EXAFS fitting and XANES and XES calculations were obtained by means of DFT geometry optimization. Clusters containing double 6-member-ring (*d6r*, Fig. S2a) and 8-member-ring (*8r*, Fig. S2b) were cut out of the bulk structure proposed by Deka *et al.*[6] (space group, R-3m; refined unit cell, a = b = 13.56 Å, c = 14.77 Å). During the optimization the framework atoms shown as sticks were frozen in order to simulate the interaction of the

rings with the framework while all the other atoms were allowed to move freely without any symmetry restriction. In order to avoid convergence problems caused by dangling bonds, hydrogen atoms were added to the terminal oxygens (not shown).

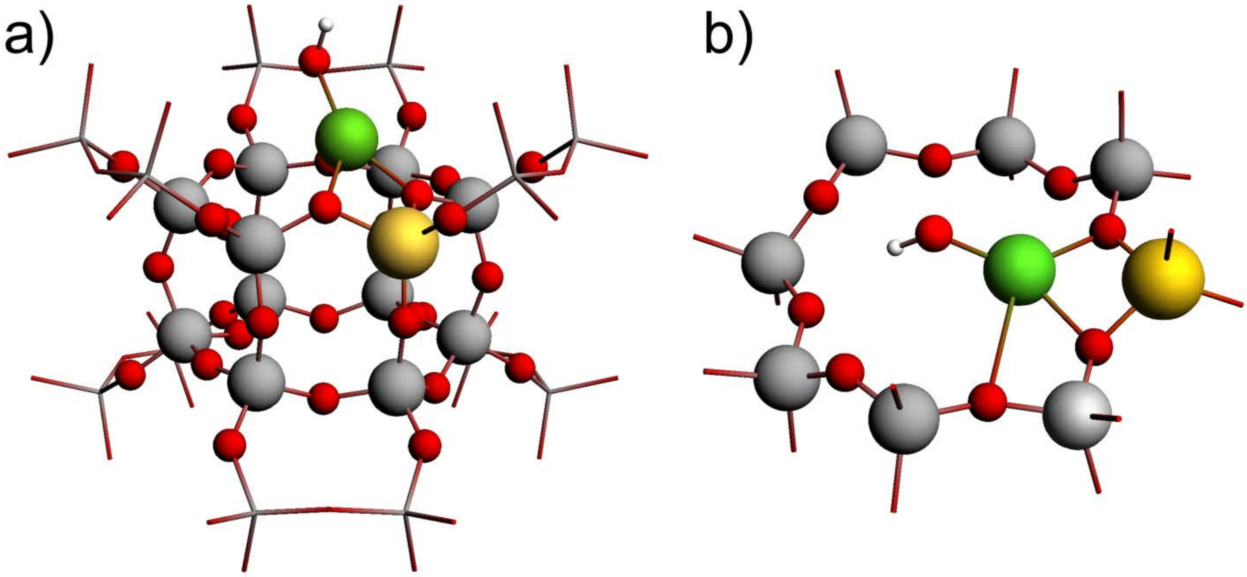

Fig. S2. Resulting cluster models for the cases of (a) double 6-member-ring and (b) 8-member ring. In both cases the OH-containing structures are presented. Color code: orange – Al, green – Cu, grey – Si, red – O, white – H. Coordinates of the atoms shown as sticks were frozen during the geometry optimization.

Geometry optimization was carried out with the ADF2012 software.[7, 8] Slater-type TZ2P basis sets were employed for all atoms together with the frozen core approximation (up to 2p frozen for Cu, Si and Al, 1s frozen for O).[9] The latter should not affect the quality of the results since the core orbitals are not strongly affected by chemical bonding, but allows to save a remarkable amount of CPU time. In order to account for exchange correlation effects, PBE functional was chosen.[10] Relativistic effects, although small, may be not negligible in this system of light elements: they were dealt with by means of the scalar ZORA approximation.[11] Convergence criteria were set to 0.001 Hartree, 0.001 Hartree/Å and 0.01 Å for energy, gradients and bond distances respectively.

1.4.2 EXAFS fitting procedure

The fits of the EXAFS spectra measured at 400 °C on the Cu-SSZ-13 catalyst stabilized after O_2- and He-activation were performed in R-space in the $\Delta R = 1.0 – 3.2$ Å range, on the FT of the k^2-weigthed $\chi(k)$ EXAFS spectra performed in the 2.4 – 12.4 $Å^{-1}$ range, resulting in 14 independent points ($2\Delta k\Delta R/\pi = 14$). Phases and amplitudes have been calculated by FEFF6 code[12, 13] using the Arthemis software from the Demeter package[5].
The different possible structures obtained from DFT optimization of Cu(II) and Cu(I) sites in the *d6r* and *8r* units of the SSZ-13 zeolite has been adopted as an input for the FEEF calculations. The experimental EXAFS spectra were fitted including in the fitting model only single-scattering (SS) paths, which represent the dominant contribution in the R-space interval of interest. To limit the number of optimized variables, all the SS paths included in the fitting model have been optimized with the same passive amplitude reduction factor (S_0^2) and with the same energy shift parameter (ΔE). As discussed in more details below, an equivalent shell model was employed to parameterize the SS paths obtained from all the candidate DFT geometries tested, to obtain a more reliable identification of the major Cu sites in the activated catalyst, as well as an experimental validation and further structural refinement of the most likely theoretical models. The number of parameters employed in the fits varied from 8 to 10, depending on the different input geometries. The quality of the fits was quantified by the R-factor parameter (perfect agreement between experimental and theoretical curve for R-factor = 0, unsatisfactory fit typically for R-factor > 0.05) computed by the Arthemis software for each EXAFS fit.
Figure S3 reports a schematic representation of the typical shell structure around the Cu-center employed to fit the EXAFS spectra of activated Cu-SSZ-13 on the basis of the different DFT models.

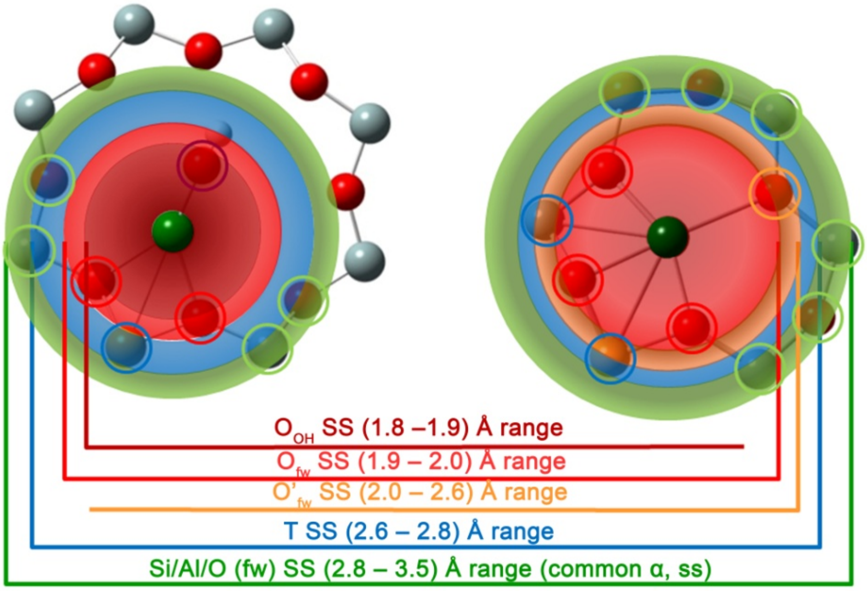

Figure S3. Common fitting model employed for the analysis of the EXAFS spectra of activated Cu-SSZ-13, aiming to discriminate between the different possible coordination environments for Cu(II) and Cu(I) sites provided by computational analysis using DFT.

A first shell of framework O_{fw} atoms is always present, and the related SS paths were parameterized with a common radial shift ($\Delta R_{O(fw)}$) applied to the individual DFT-optimized bond distances and Debye-Waller (DW) factor ($\sigma^2_{O(fw)}$). For the Cu(II) models considering a $[CuOH]^+$ complex in proximity of a (**1Z**$^-$) site, the SS involving the O(OH) atom is parameterized with specific $\Delta R_{O(OH)}$ and $\sigma^2_{O(OH)}$, to properly account for its shorter bond distance foreseen by DFT and for the different chemical nature of this ligand with respect to the O_{fw} atoms.

Some models in the *d6r* are characterized by two sub-shells of oxygen atoms. In these cases, an additional radial shift parameter $\Delta R_{O'(fw)}$ have been employed for the SS paths involving the second sub-shell atoms, including one or two O'_{fw} at distances in the (2.0 –2.6) Å range from the Cu absorber. The DW for these paths has been refined to the same value used for the contribution involving the first shell framework oxygens: $\sigma^2_{O'(fw)} = \sigma^2_{O(fw)}$.

Subsequently, a shell of one or two T (Al and Si, indistinguishable by EXAFS) atoms is always present, with distances from the Cu center in the (2.6 – 2.8) Å range: the SS paths involving these atoms have been parameterized with a common radial shift (ΔR_T) and DW factor (σ^2_T).

Finally, a lower-level parametrization strategy has been adopted for the SS paths which involve the most far Si, Al and O atoms of the *d6r* and *8r* (2.8 –3.5 Å distance range from the Cu center), providing a minor but not negligible contribution to EXAFS signal. These SS paths were modeled considering a common contraction/expansion factor α_{fw} and DW factor σ^2_{fw} increasing as the square root of the distance $R_{eff,i}$ of the ith scattering atom from the absorber ($\Delta R_{fw,i} = \alpha_{fw} R_{eff,i}$, $\sigma^2_{fw,i} = ss_{fw} (R_{eff,i})^{1/2}$).

The number of parameters varied from 8 to 10, depending on the specific DFT-optimized geometries employed as starting guess, being always below the number of employed independent points ($2\Delta k\Delta R/\pi > 14$).

1.4.3 XANES and XES simulations

XES and XANES spectra were simulated using the molecular orbitals calculated by ADF. After geometry optimization, an all-electron single point calculation was performed for each structure using very large QZ4P basis set in order to obtain high-quality data for both core and valence orbitals. Then the resulting molecular orbitals were projected onto the cubic grid centered around the absorbing atom. Grid dimensions were 1 Å in each direction and totally it comprised 51^3=132651 points. The Cu valence-to-core XES and K-edge XANES spectra were obtained by numerical volume integration of the 1s core orbitals with the occupied and unoccupied valence orbitals respectively within the dipole approximation. Integration was performed by an in-house software developed by Dr. G. Smolentsev and previously used for XES[14] and XANES simulations.[15, 16]

2 XAS of hydrated Cu-SSZ-13

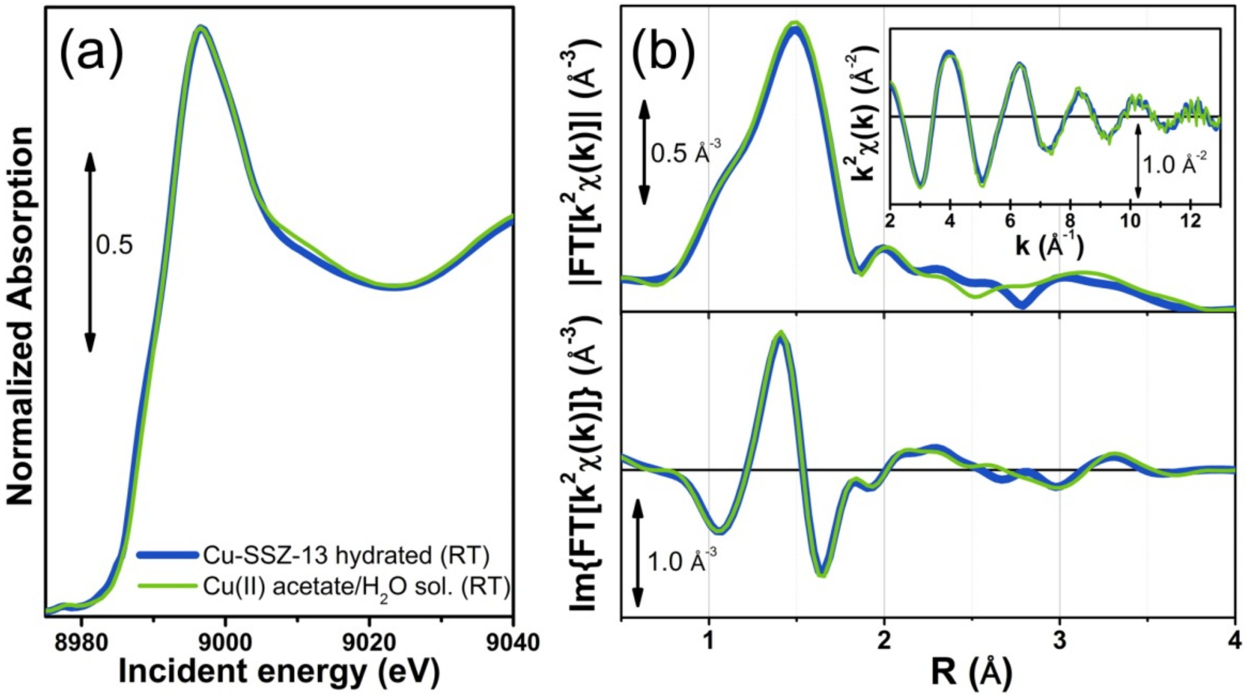

Fig. S4. Comparison between XAS data collected on hydrated Cu-SSZ-13 (blue thicker lines) and a 50 mM solution of Cu(II) acetate in water (green thinner lines), both collected at room temperature at the BM23 beamline of the ESRF. (a) Cu K-edge XANES spectra. (b) EXAFS spectra: the magnitude and the imaginary part of the FT performed over the k^2-weighted $\chi(k)$ curves shown in the inset, in the (2.4 – 12.4) $Å^{-1}$ range, are reported in the top and bottom panel respectively.

3 Reversibility of the Cu(I) ↔ Cu(II) redox chemistry and extra-ligand loss process upon high-temperature gas-flow switching

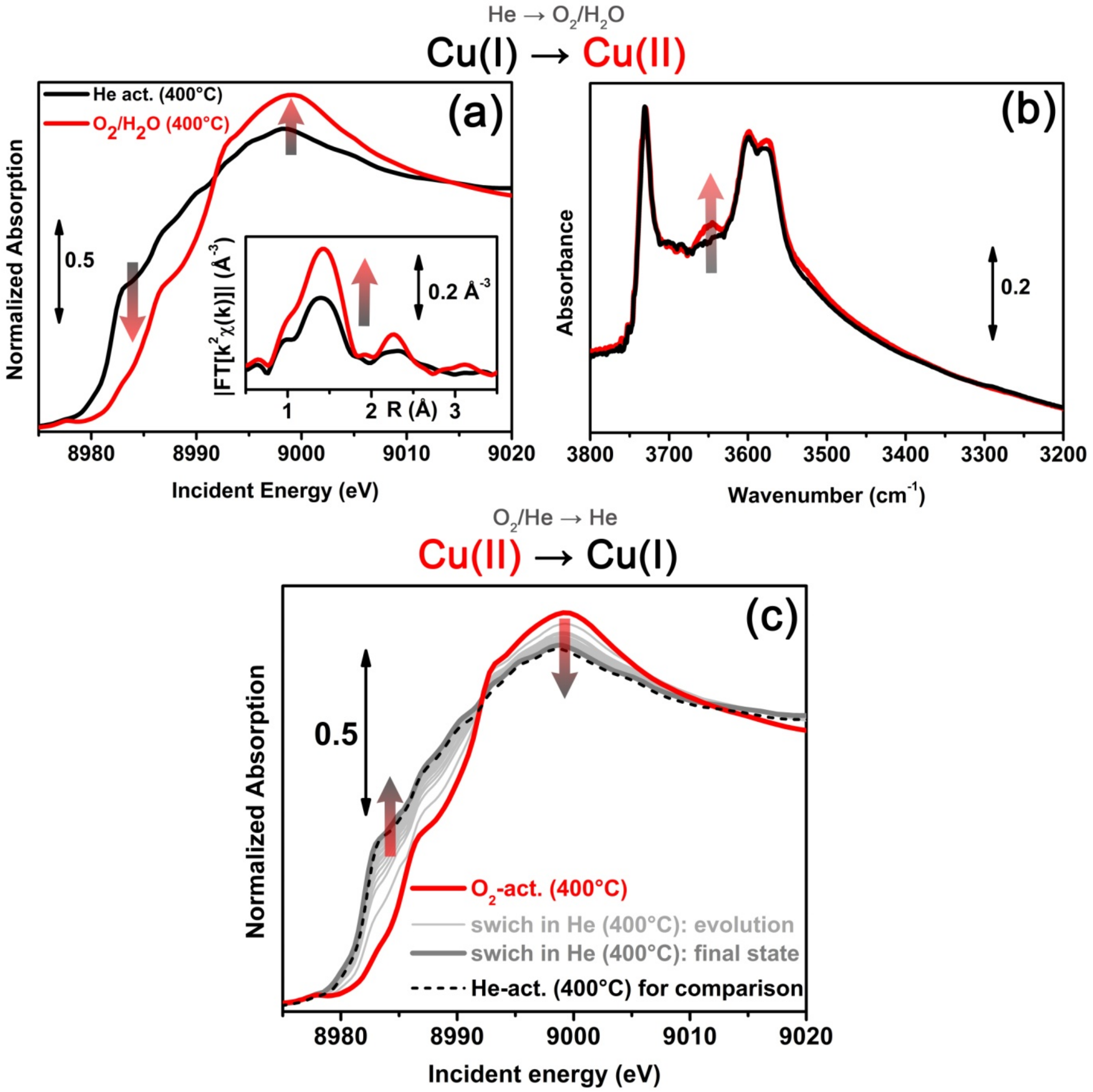

Fig. S5. (a), (b) Effect of O_2/H_2O interaction (red curves) with He-activated Cu-SSZ-13 (black curves) at 400 °C; (a) XANES (main panel) indicates that Cu(I) can be easily re-oxidized to Cu(II), while FT EXAFS spectra (inset) evidence an increase in the first-shell coordination number of Cu. The overall XAS features are equivalent to that observed for the final state achieved after O_2-activation; (b) FTIR spectroscopy shows the appearance of the 3656 cm^{-1} feature, indicating that $[CuOH]^+$ formation occurs upon O_2/H_2O interaction with Cu(I) sites. (c) Effect of switching to pure He flux at 400 °C (grey curves) starting from the O_2-activated Cu-SSZ-13 (red line), as monitored by XANES: waiting in He, the typical Cu(I) XANES features are progressively restored, up to a final state fully equivalent to that observed upon He-activation (black dashed line, reported for comparison).

4 Full report on DFT-optimized geometries

The different DFT models have been named according to the nomenclature reported in Scheme S1.

	CHA unit	number of Al atoms per unit	OH extra-ligand (when present)	disposition of 2 Al atoms (when present)
options	*d6r*	*1Al*	–	–<br>***#1*** = Al–(O–Si)–O–Al<br>***#2*** = Al–(O–Si)$_2$–O–Al<br>***#3*** = Al–(O–Si)$_3$–O–Al
	8r	*2Al*	*OH*	

Scheme S1. Nomenclature adopted for the different DFT-optimized models describing Cu(II) and Cu(I) sites in the *d6r* and *8r* units of the CHA framework.

In particular, the name of each model is composed by the sequence of the following text fields:

- CHA unit of interest: ***d6r*** or ***8r***
- number of Al atoms in the unit of interest: ***1Al*** or ***2Al***
- OH extra-ligand: ***OH*** (when present) or *nothing* (when not present)
- additional field for ***2Al*** models only, to distinguish the possible dispositions of two Al atoms in the same unit: ***#1*** = Al–(O–Si)–O–Al or ***#2*** = Al–(O–Si)$_2$–O–Al or ***#3*** = Al–(O–Si)$_3$–O–Al.

Table S1 reports the individual DFT-optimized distances from Cu for the nearest atoms of the *d6r* and *8r* units of the SSZ-13 zeolite as well as the average distances for the principal shells of atomic neighbors surrounding the Cu centers

Table S1. Detailed report on individual and average Cu bond distances obtained in DFT-optimized geometries for Cu(II) and Cu(I) sites in *d6r* and *8r* units of the Cu-SSZ-13 catalyst. For the atom labeling code please refer to Fig. 5 in the main text.

	Cu(II) sites							Cu(I) sites	
Bond length (Å)	***d6r1Al OH***	***d6r2Al #1***	***d6r2Al #2***	***8r1Al OH***	***8r2Al #1***	***8r2Al #2***	***8r2Al #3***	***d6r1Al***	***8r1Al***
Cu–O1$_{OH}$	1.774	–	–	1.757	–	–	–	–	–
Cu–O1$_{fw}$	1.957	1.933	1.947	1.946	2.070	1.934	1.918	1.915	1.948
Cu–O2$_{fw}$	2.008	2.068	2.069	2.036	1.952	1.932	1.959	–	1.977
Cu–O3$_{fw}$	–	1.920	2.095	–	2.067	–	–	2.000	–
Cu–O4$_{fw}$	–	–	2.003	–	–	–	–	–	–
<Cu–O$_{fw}$>	1.982	1.974	2.028	1.990	2.030	1.933	1.938	1.957	1.962
Cu–O'1$_{fw}$	–	2.393	–	–	–	–	–	2.451	–
Cu–O'2$_{fw}$	–	–	–	–	–	–	–	2.571	–
<Cu–O'$_{fw}$>	–	2.393	–	–	–	–	–	2.511	–
Cu–T1	2.782	2.752	2.805	2.690	2.631	2.681	2.707	2.792	2.709
Cu–T2	–	2.677	2.805	–	2.679	–	–	2.830	–
<Cu–T>	2.782	2.714	2.805	2.735	2.655	2.681	2.707	2.811	2.709

After DFT geometry optimization, we found that the local environment of the $[CuOH]^+$ complex is rather similar in the *d6r* and *8r* units. Cu(II) cations are coordinated in a distorted planar-trigonal geometry to the (OH) group and to two framework O atoms, with bond length almost equivalent in the two units (shorter for the OH group: Cu–O$_{OH}$ ~ 1.78 Å, with respect to Cu–O$_{fw}$ distances of ~ 1.99 Å). In terms of radial bond distance from the Cu center, the two geometries only differ due to a ~ 0.1 Å elongation of the Cu–T distance in the *d6r* with respect to the *8r*. Moreover, in the *d6r* the complex points out from the plane of the ring, with an almost perfect collinearity along the T1-Cu-O$_{OH}$ axis. Conversely, in the *8r* the $[CuOH)]^+$ complex lies more close to the plane of the ring, with the T1-Cu-O$_{OH}$ angle slightly decreased from 180°.

In correspondence of (**2Z**$^-$) sites in the *d6r*, the Cu(II) cation is hosted in the plane of the ring, only slightly shifted from its centers towards the nearest Al atom. Cu is set next to four O$_{fw}$ atoms, in a highly distorted square-planar-like

geometry. In the Al–(O–Si)–O–Al configuration (*d6r2Al#1* model), three out of four Cu–O_{fw} bond lengths are almost equivalents, averaging at ~ 1.97 Å, while the fourth O atom is located at ~ 2.39 Å, justifying its inclusion in a different coordination shell for EXAFS analysis (O'$_{fw}$ shell). Conversely, in the Al–(O–Si)$_2$–O–Al configuration (*d6r2Al#2* model), all the four Cu–O_{fw} bonds fall in the (1.95 – 2.00) Å range, averaging at ~ 2.03 Å. From both this models, two framework T atoms (1 Al and one Si atom, indistinguishable by XAS due to the similar atomic number *Z*) are located at about the (2.70–2.80) Å from Cu.
Considering Cu(II) ions stabilized next to (**2Z‾**) sites in the *8r*, a coordination environment very similar to that found in model *d6r2Al#1* is obtained for the Al–Si–Al configuration (three nearest-neighbors O atoms at ~ 2.03 Å, and two T atoms, 1 Al and one Si, at ~ 2.65 Å from Cu). On the contrary, considering both Al–(O–Si)$_2$–O–Al and Al–(O–Si)$_3$–O–Al configurations (models *8r2Al#2 and 8r2Al#3*, respectively), less coordinated Cu-sites are found, with a first shell consisting of two O atoms at ~ 1.93 Å from the cation, which is located in proximity of 1 of the two Al atoms of the ring. In these latter cases, the separation between the two **Z‾** sites in the larger *8r* is likely enough to avoid a combined influence of the two Al atoms on the Cu cation. Thus, it is not surprising to find very similar coordination geometry for Cu(I) sites realized stabilizing the "bare" Cu cation next to a (**1Z‾**) sites in the *8r*, as in model *8r1Al*. Differently from what observed for the Cu(II) models including the (OH) extra-ligand, the Cu(I) site coordination geometry is significantly modified in the *d6r* sub-unit, with respect to what observed in the *8r*. Indeed, here Cu is shifted towards the centre of the ring, surrounded by two sub-shells constituted by two O atoms each, at ~ 1.96 Å (O_{fw}) and ~ 2.51 Å (O'$_{fw}$). A second shell including two T atoms is then found at ~ 2.71 Å from the Cu center.

5 Full report on EXAFS fitting results on O_2-activated Cu-SSZ-13 – Cu(II) sites

Table S2. Detailed report on the best-fit parameters optimized in the EXAFS fit of the k^2-weighted spectrum of O_2-activated Cu-SSZ-13 (data collection at 400 °C), employing as starting guess in the fitting procedure the DFT-optimized geometries for Cu(II)-sites in the *d6r* unit. The fit was performed in R-space, in the range (1.0 – 3.2) Å, employing the k-range (2.4 – 12.4) $Å^{-1}$ for the FT (14 independents points).

Cu(II) sites (O_2-activated Cu-SSZ-13) in *d6r* unit			
	d6r1AlOH	***d6r2Al#1***(*)	***d6r2Al#2***(**)
geometry	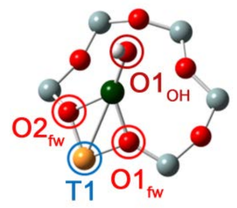	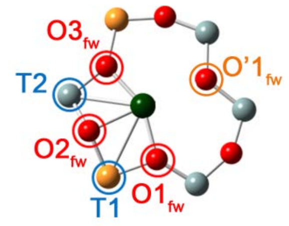	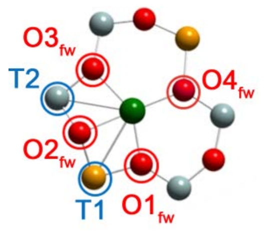
S_0^2	0.99 ± 0.09	1.00 ± 0.06	0.77 ± 0.06
ΔE (eV)	– 0.3 ± 0.9	– 1.4 ± 0.9	– 1.4 ± 0.9
R-factor	0.00350	0.00304	0.00440
N_{par}	10	9	8
R_{OH} (Å)	1.87 ± 0.03	–	–
σ^2_{OH} ($Å^2$)	0.004 ± 0.002	–	–
N_{OH}	1	–	–
$R_{O(fw)}$(Å)	1.98 ± 0.02	1.943 ± 0.006	1.923 ± 0.007
$\sigma^2_{O(fw)}$ ($Å^2$)	0.007 ± 0.004	0.005± 0.001	0.006± 0.001
$N_{O(fw)}$	2	3	4
$R_{O'(fw)}$ (Å)	–	2.63 ± 0.01	–
$\sigma^2_{O'(fw)}$ ($Å^2$)	–	0.005± 0.001	–
$N_{O'(fw)}$	–	1	–
R_T (Å)	2.72 ± 0.02	2.71 ± 0.02	2.70 ± 0.01
σ^2_T ($Å^2$)	0.008 ± 0.002	0.009± 0.001	0.014± 0.002
N_T	1	2	2
α_{fw}	– 0.04 ± 0.02	– 0.03 ± 0.11	– 0.03 ± 0.01
σ^2_{fw} ($Å^2$)	0.008 ± 0.005	0.05 ± 0.5	0.023 ± 0.06
N_{fw}	4	6	6

(*) Strong antiphase effects between O_2 and T shells

(**) Critical S_0^2 values

Table S3. Detailed report on the best-fit parameters optimized in the EXAFS fit of the k^2-weighted spectrum of O_2-activated Cu-SSZ-13 (data collection at 400 °C), employing as starting guess in the fitting procedure the DFT-optimized geometries for Cu(II)-sites in the *8r* unit. The fit was performed in R-space, in the range (1.0 – 3.2) Å, employing the k-range (2.4 – 12.4) $Å^{-1}$ for the FT (14 independents points).

Cu(II) sites (O_2-activated Cu-SSZ-13) in *8r* unit				
	8r1AlOH	***8r2Al#1***	***8r2Al#2***(**)	***8r2Al#3***(**)
geometry	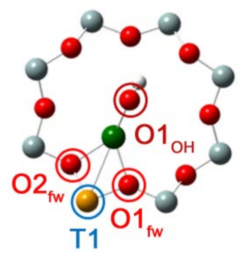	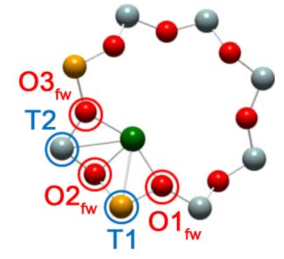	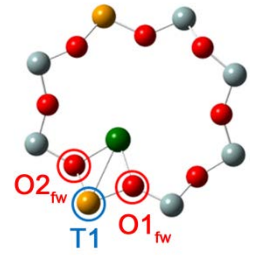	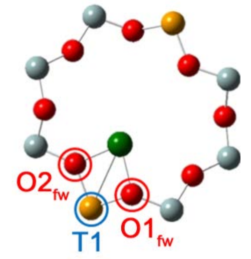
S_0^2	0.96 ± 0.07	1.1 ± 0.1	1.6 ± 0.1	1.6 ± 0.1
ΔE (eV)	– 1.8 ± 0.6	1 ± 1	– 2.6 ± 0.8	0.3 ± 0.8
R-factor	0.00220	0.01751	0.00413	0.00639
N_{par}	9	8	8	8
R_{OH} (Å)	1.85 ± 0.02	–	–	–
σ^2_{OH} ($Å^2$)	0.004 ± 0.002	–	–	–
N_{OH}	1	–	–	–
$R_{O(fw)}$(Å)	1.97 ± 0.01	1.936 ± 0.009	1.928 ± 0.006	1.933 ± 0.006
$\sigma^2_{O(fw)}$ ($Å^2$)	0.004 ± 0.002	0.007± 0.002	0.010± 0.001	0.009± 0.001
$N_{O(fw)}$	2	3	2	2
$R_{O'(fw)}$ (Å)	–	–	–	–
$\sigma^2_{O'(fw)}$ ($Å^2$)	–	–	–	–
$N_{O'(fw)}$	–	–	–	–
R_T (Å)	2.72 ± 0.01	2.68 ± 0.02	2.73 ± 0.02	2.72 ± 0.02
σ^2_T ($Å^2$)	0.008 ± 0.001	0.017± 0.003	0.014± 0.002	0.015± 0.003
N_T	1	2	1	1
α_{fw}	– 0.01 ± 0.01	– (***)	– 0.01 ± 0.02	– 0.02 ± 0.01
σ^2_{fw} ($Å^2$)	0.022 ± 0.005	– (***)	0.019 ± 0.008	0.013 ± 0.004
N_{fw}	4	– (***)	4	4

(*) Strong antiphase effects between O_2 and T shells
(**) Critical S_0^2 values
(***) For the *8r2Al#1* model the SS path involving the fw shell fall outside from the R-space fitting range

6 EXAFS spectra for a low Cu-loading Cu-SSZ-13 sample after O_2-activation @400 °C

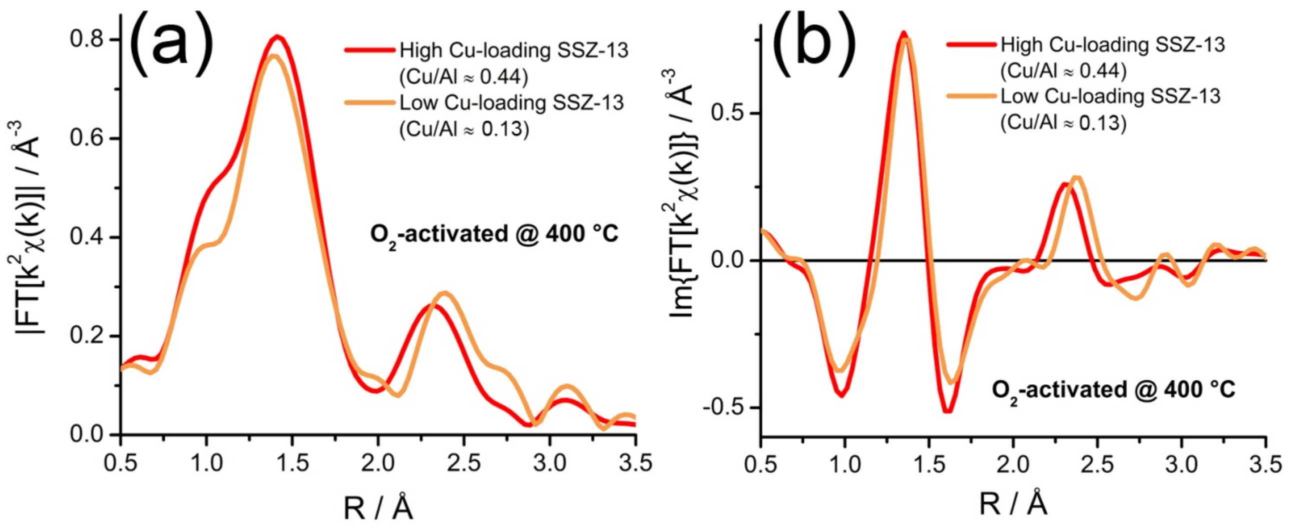

Fig. S6. Preliminary EXAFS measurements performed at the BM23 beamline of the ESRF on a low-Cu-loading Cu-SSZ-13 sample (Cu/Al = 0.13) after O_2-activation (data collection at 400 °C), in the same experimental conditions employed in the characterization of the high-Cu-loading sample discussed in the main text. The FT of k^2-weighted $\chi(k)$ spectra (magnitude and imaginary part of the FT are reported in part (a) and (b), respectively) collected for the low-Cu-loading sample (orange lines) are compared with that measured for the high-Cu-loading sample (red lines), already reported and analyzed in the main text. It is important to note that, due to the lower concentration of the absorber atoms, the spectrum of the low-Cu-loading sample is affected by a lower S/N ratio. However, for the sake of comparison, the reported FT spectra has been calculated using the same (2.4 – 12.4) $Å^{-1}$ k-range.

7 References

1. F. Giordanino, P. N. R. Vennestrom, L. F. Lundegaard, F. N. Stappen, S. L. Mossin, P. Beato, S. Bordiga and C. Lamberti, *Dalton Trans.*, 2013, **42**, 12741-12761.
2. F. Giordanino, E. Borfecchia, K. A. Lomachenko, A. Lazzarini, G. Agostini, E. Gallo, A. V. Soldatov, P. Beato, S. Bordiga and C. Lamberti, *J. Phys. Chem. Lett.*, 2014, **5**, 1552-1559.
3. S. Bordiga, E. Groppo, G. Agostini, J. A. van Bokhoven and C. Lamberti, *Chem. Rev.*, 2013, **113**, 1736-1850.
4. C. Lamberti, S. Bordiga, F. Bonino, C. Prestipino, G. Berlier, L. Capello, F. D'Acapito, F. Xamena and A. Zecchina, *Phys. Chem. Chem. Phys.*, 2003, **5**, 4502-4509.
5. B. Ravel and M. Newville, *J. Synchrotron Rad.*, 2005, **12**, 537-541.
6. U. Deka, A. Juhin, E. A. Eilertsen, H. Emerich, M. A. Green, S. T. Korhonen, B. M. Weckhuysen and A. M. Beale, *J. Phys. Chem. C*, 2012, **116**, 4809-4818.
7. ADF2012, SCM, Theoretical Chemistry, Vrije Universiteit, Amsterdam, The Netherlands, http://www.scm.com.
8. G. te Velde, F. M. Bickelhaupt, E. J. Baerends, C. F. Guerra, S. J. A. Van Gisbergen, J. G. Snijders and T. Ziegler, *J. Comput. Chem.*, 2001, **22**, 931-967.
9. E. van Lenthe and E. J. Baerends, *J. Comput. Chem.*, 2003, **24**, 1142-1156.
10. J. P. Perdew, K. Burke and M. Ernzerhof, *Phys. Rev. Lett.*, 1996, **77**, 3865-3868.
11. E. van Lenthe, A. Ehlers and E. J. Baerends, *J. Chem. Phys.*, 1999, **110**, 8943-8953.
12. S. I. Zabinsky, J. J. Rehr, A. Ankudinov, R. C. Albers and M. J. Eller, *Phys. Rev. B*, 1995, **52**, 2995-3009.
13. J. J. Rehr and R. C. Albers, *Rev. Mod. Phys.*, 2000, **72**, 621-654.
14. G. Smolentsev, A. V. Soldatov, J. Messinger, K. Merz, T. Weyhermuller, U. Bergmann, Y. Pushkar, J. Yano, V. K. Yachandra and P. Glatzel, *J. Am. Chem. Soc.*, 2009, **131**, 13161-13167.
15. I. Alperovich, G. Smolentsev, D. Moonshiram, J. W. Jurss, J. J. Concepcion, T. J. Meyer, A. Soldatov and Y. Pushkar, *J. Am. Chem. Soc.*, 2011, **133**, 15786-15794.
16. K. A. Lomachenko, C. Garino, E. Gallo, D. Gianolio, R. Gobetto, P. Glatzel, N. Smolentsev, G. Smolentsev, A. V. Soldatov, C. Lamberti and L. Salassa, *Phys. Chem. Chem. Phys.*, 2013, **15**, 16152-16159.
